# Morphine-Induced Modulation of Endolysosomal Iron Mediates Upregulation of Ferritin Heavy Chain in Cortical Neurons

**DOI:** 10.1523/ENEURO.0237-19.2019

**Published:** 2019-07-29

**Authors:** Bradley Nash, Kevin Tarn, Elena Irollo, Jared Luchetta, Lindsay Festa, Peter Halcrow, Gaurav Datta, Jonathan D. Geiger, Olimpia Meucci

**Affiliations:** 1 Department of Pharmacology and Physiology, Drexel University College of Medicine, Philadelphia, PA 19102; 2 Department of Biomedical Sciences, University of North Dakota School of Medicine and Health Sciences, Grand Forks, ND 58203; 3 Department of Microbiology and Immunology, Drexel University College of Medicine, Philadelphia, PA 19102

**Keywords:** dendritic spine, endolysosome, ferritin, morphine, neuroHIV, neuron

## Abstract

HIV-associated neurocognitive disorders (HAND) remain prevalent and are aggravated by µ-opioid use. We have previously shown that morphine and other µ-opioids may contribute to HAND by inhibiting the homeostatic and neuroprotective chemokine receptor CXCR4 in cortical neurons, and this novel mechanism depends on upregulation of the protein ferritin heavy chain (FHC). Here, we examined the cellular events and potential mechanisms involved in morphine-mediated FHC upregulation using rat cortical neurons of either sex in vitro and in vivo. Morphine dose dependently increased FHC protein levels in primary neurons through µ-opioid receptor (µOR) and Gαi-protein signaling. Cytoplasmic FHC levels were significantly elevated, but nuclear FHC levels and FHC gene expression were unchanged. Morphine-treated rats also displayed increased FHC levels in layer 2/3 neurons of the prefrontal cortex. Importantly, both *in vitro* and *in vivo* FHC upregulation was accompanied by loss of mature dendritic spines, which was also dependent on µOR and Gαi-protein signaling. Moreover, morphine upregulated ferritin light chain (FLC), a component of the ferritin iron storage complex, suggesting that morphine altered neuronal iron metabolism. Indeed, prior to FHC upregulation, morphine increased cytoplasmic labile iron levels as a function of decreased endolysosomal iron. In line with this, chelation of endolysosomal iron (but not extracellular iron) blocked morphine-induced FHC upregulation and dendritic spine reduction, whereas iron overloading mimicked the effect of morphine on FHC and dendritic spines. Overall, these data demonstrate that iron mediates morphine-induced FHC upregulation and consequent dendritic spine deficits and implicate endolysosomal iron efflux to the cytoplasm in these effects.

## Significance Statement

Clinical studies suggest that opioid use exacerbates HIV-associated neurocognitive disorders (HAND), but the mechanisms by which opioids contribute to HAND are not completely understood. This work demonstrates that morphine reduces the density of mature dendritic spines of cortical neurons through a novel mechanism involving neuronal iron metabolism. We showed that morphine induces efflux of endolysosomal iron to the cytoplasm, resulting in a post-transcriptional upregulation of ferritin heavy chain (FHC). FHC upregulation inhibits the homeostatic and neuroprotective CXCL12/CXCR4 chemokine signaling axis, producing dendritic spine deficits. Importantly, morphine’s actions on FHC and dendritic spines are blocked by chelation of endolysosomal iron, suggesting that endolysosomal iron stores are key components of dendritic injury in opioid using HAND patients.

## Introduction

HIV-associated neurocognitive disorder (HAND) pathology has significantly improved with the advent of antiretroviral therapies (ART). Patients managed successfully with ART rarely present with the most severe form of HAND, HIV-associated dementia (McArthur et al., 2010). However, less severe forms of HAND persist in the ART era, and the prevalence of HAND is increasing as the ART-treated patient population ages (Saylor et al., 2016). Previous therapies designed to treat HAND have failed to show efficacy in clinical trials (McGuire et al., 2014), demonstrating the need to discover new drug targets and develop new adjuvant therapies.

Many patients with HIV are infected by sharing opioid injection equipment with other infected individuals. Additionally, clinical and preclinical studies suggest that µ-opioid use worsens HAND symptoms (Bandaru et al., 2011; Byrd et al., 2011; Guo et al., 2012; Fitting et al., 2014; Pitcher et al., 2014; El-Hage et al., 2015; Xu and Fitting, 2016). This may be due, at least in part, to µ-opioid receptor (µOR)-mediated inhibition of the CXCL12/CXCR4 chemokine signaling axis in cortical neurons (Sengupta et al., 2009). For example, CXCL12/CXCR4 signaling in the CNS regulates neural progenitors migration and differentiation (Kokovay et al., 2010; Wang et al., 2011), cell cycle entry (Khan et al., 2003, 2008), glutamatergic signaling (Nicolai et al., 2010; Di Prisco et al., 2016), GABAergic neurotransmission (Guyon, 2014; Wu et al., 2017), neuronal excitability (Guyon and Nahon, 2007; Shepherd et al., 2013), and dendritic spine density (Pitcher et al., 2014; Festa et al., 2015). CXCL12/CXCR4 signaling is also neuroprotective in toxic environments (Meucci et al., 1998; Shepherd et al., 2013; Chiazza et al., 2018).

Our previous studies show that µ-opioid-mediated inhibition of neuronal CXCR4 requires upregulation of ferritin heavy chain (FHC) protein (Sengupta et al., 2009; Pitcher et al., 2014). FHC interacts with the CXCR4 protein complex, producing a long-lasting inhibition of the receptor that is distinct from more common opioid-chemokine cross-regulatory mechanisms including heterologous desensitization (Chen et al., 2007) and receptor dimerization (Pello et al., 2008; Nash and Meucci, 2014). Upregulation of FHC is also required for µ-opioid induced dendritic spine deficits, as FHC knockdown completely prevents morphine’s ability to reduce spine density in primary neuronal cultures (Pitcher et al., 2014). As dendritic spines in select brain regions are critical mediators of learning and memory (Morrison and Baxter, 2012), µ-opioids may contribute to HAND pathology by reducing spine density in these regions. Indeed, FHC protein expression in prefrontal cortex neurons correlates with cognitive impairment in HAND patients (Pitcher et al., 2014).

In addition to its role in CXCR4 regulation, FHC is well known as a subunit of the iron storage protein ferritin (Knovich et al., 2009). As such, FHC protein expression is regulated by intracellular labile iron levels and iron-related stimuli including oxidative damage and inflammatory cytokines (Torti and Torti, 2002; Wilkinson et al., 2003). Furthermore, clinical studies show that cognitive deficits in HAND are associated with increased levels of iron transport proteins in CSF (Kallianpur et al., 2019), and that CSF iron and FHC levels are associated with CSF viral load and plasma virus detectability, respectively (Patton et al., 2017). Therefore, increased CNS iron levels in HAND may drive the production of FHC, which could then lead to dendritic spine deficits and cognitive impairment. However, the mechanism by which morphine upregulates neuronal FHC is unclear. Since FHC is classically regulated by iron levels, and iron dysregulation is associated with HAND symptoms, we suspected that morphine may also upregulate FHC by modulating neuronal iron metabolism. Intriguingly, we found that morphine causes iron efflux from endolysosomes to the labile iron pool in the cytoplasm, which is required for FHC upregulation in cortical neurons. These results imply that opioid use and HIV infection may share a convergent mechanism leading to cognitive impairment, thus targeting brain-iron metabolism may be an effective approach for future HAND therapeutics.

## Materials and Methods

### Cell cultures

Primary neuronal cultures were prepared with cortical tissue from mixed male and female Holtzman E17 rat embryos, as we have previously described (Sengupta et al., 2009; Pitcher et al., 2014; Festa et al., 2015). We prepared two different types of primary neuronal cultures, which allowed for maintenance of cortical neurons in the presence or absence of glia. Most experiments used primary cortical neurons grown in the absence of glia (neurobasal cultures), as originally described by (Brewer et al., 1993). Briefly, dissected cortical neurons were plated in neurobasal medium (Gibco 21103) containing 2% heat inactivated donor equine serum (Hyclone SH3007403HI), 2% B-27 supplement 50X (Gibco 17504), 0.5 mM GlutaMAX (Gibco 35050), 25 µM L-glutamic acid (Tocris 0218), and 50 µg/ml gentamicin (Gibco 15750) for the first 4 h of culture. Following a wash with neurobasal medium, culture media was changed to the same neurobasal medium formula without donor equine serum. On fifth day *in vitro* (DIV 5), medium was replaced with neurobasal, containing 2% B-27, 0.5 mM GlutaMAX, and 50 µg/ml gentamicin; the medium was changed every 4 d for the life of the culture.

Select experiments used primary cortical neurons cultured in the presence of a glial feeder layer (bilaminar cultures), as we described previously (Shimizu et al., 2011a). Bilaminar cultures show how the presence of glia affects neurons in various treatment conditions while still enabling direct investigation of neurons, as the glial feeder layer can be removed before lysis. Dissected primary neurons were plated in DMEM (Gibco 11995) with 10% heat inactivated donor equine serum (Hyclone SH3007403HI) for 4 h. Following a wash with DMEM, culture media was changed to DMEM containing 1% N2.1 supplement (Gibco 17502), and 0.5 mg/ml ovalbumin (Sigma A2512). One-half of the culture medium was replaced every 7 DIV. DIV 9–14 neurons were used for all experiments except dendritic spine studies, which used DIV 21 cortical neurons.

For endolysosomal pH and iron measurement studies with FeRhoNox-1 and phen green SK, E18 rat cortical neurons were purchased from BrainBits LLC (FSDECX1M) and cultured in neurobasal medium as described above, with half-media changes every 4 d.

Hippocampal neurons were prepared and cultured as described in (Bae et al., 2014), and were used for experiments at 8–15 DIV, while glioblastoma (U87) cells were cultured in DMEM (Invitrogen) containing 10% fetal bovine serum and 1% penicillin/streptomycin (Invitrogen), and were grown to confluence in a 5% CO_2_ incubator at 37°C. U87 cells were used up to their tenth passage.

### *In vitro* treatments

Morphine sulfate (Sigma M8777) was dissolved in ultrapure water, passed through 0.2 µM syringe filters into sterile Eppendorf tubes, and frozen at –20°C in the dark until use. *In vitro* experiments also used CTAP (Tocris 1560), naloxone (Sigma N7758), pertussis toxin (PTX; Sigma P7208), tumor necrosis factor α (TNFα; R&D systems 210-TA), ferric ammonium citrate (FAC; Sigma F5879), deferoxamine mesylate (DFO; Sigma D9533), diethylenetriaminepentaacetic acid (DTPA; Sigma D6518), and phenanthroline (Sigma 131377), which were prepared according to the manufacturer’s instructions. When neuronal cultures were appropriately aged for experiments, fresh stock solutions were diluted to working concentrations in culture medium.

### Western blots

Total protein extracts, and cytoplasmic/nuclear extracts were obtained by standard protocols, as previously reported (Khan et al., 2008; Sengupta et al., 2009). Total protein extracts of cells or tissues were obtained in triple-detergent lysis buffer (150 mM NaCl, 50 mM Tris, 0.5% Na deoxycholate, 0.1% SDS, 10 mM Na_4_P_2_O_7_, 5 mM EDTA, 1% Triton X-100, and 1 mM DTT), containing protease inhibitors (Thermo Scientific 1861278) and phosphatase inhibitors (Calbiochem 524625). Equal amounts of protein (30–40 μg) were loaded into each lane for SDS-PAGE and transferred to PVDF membranes for immunoblotting.

Cytoplasmic and nuclear proteins were separately extracted by first using a hypotonic buffer solution [20 mM Tris-HCl, pH 7.4, 10 mM NaCl, 3 mM MgCl_2_, 1 mM AEBSF (Sigma A8456), 5 µg/ml aprotinin (Sigma A4529), 5 µg/ml leupeptin (Sigma L2884), and 5 µg/ml pepstatin A (Sigma P5318)] to lyse cells without breaking the nuclei (500 µl/5 × 10^6^ cells on ice for 15 min with gentle mixing every 5 min). Then 0.05% IGEPAL CA-630 (Sigma I8896) was added to each lysate, and lysates were vortexed for 10 s. The cytoplasmic fraction was isolated as a supernatant by centrifugation (14,000 rpm, 2 min, 4°C). Nuclei pellets were washed twice with the hypotonic buffer solution to remove residual cytoplasmic contamination, then lysed using a triple detergent buffer (100 mM Tris, pH 7.4, 2 mM Na_3_VO_4_, 100 mM NaCl, 1% Triton X-100, 1 mM EDTA, 10% glycerol, 1 mM EGTA, 0.1% SDS, 1 mM NaF, 0.5% deoxycholate, 20 nM Na_4_P_2_O_7_, 1 mM AEBSF, 5 µg/ml aprotinin, 5 µg/ml leupeptin, and 5 µg/ml pepstatin A). Nuclei pellets were incubated in 50 µl of the triple detergent buffer on ice for 45 min, with vortex mixing every 10 min. Then, the lysed nuclei solutions were centrifuged (14,000 rpm, 30 min, 4°C) to obtain nuclear extracts. Cytoplasmic and nuclear extracts were stored at –80°C until use.

Antibodies used for Western blotting experiments included: anti-FHC (Cell Signaling Technology 3998, RRID:AB_1903974, 1:1000), anti-FLC (Abcam ab 69090, RRID:AB_1523609, 1:3000), anti-β-actin (Sigma-Aldrich A2066, RRID:AB_476693, 1:6000), anti-GAPDH (Cell Signaling 5174S, RRID:AB_10622025, 1:4000), and anti-histone H3 (Cell Signaling Technology 9715, RRID:AB_331563, 1:1000). Band densities were quantified with UN-SCAN-IT gel v. 6.1, RRID:SCR_017291.

### Real-time quantitative PCR

Total RNA was extracted from neurons using the RNeasy mini kit (Qiagen 74104) following instructions from the manufacturer. RNA quality and concentration were assessed with a NanoDrop ND-100 spectrophotometer (NanoDrop Technologies). cDNA was then synthesized by reverse transcription using random hexamers (Thermo Fisher N8080127) as described previously (Sengupta et al., 2009). Real-time PCR reaction was performed using TaqMan probes for the target gene FHC and housekeeping gene GAPDH (Applied Biosystems; Rn00820640_g1 and 4352338E) in Taqman Gene Expression Master Mix (Thermo Fisher 4369016). Samples were run in triplicate in 96-well reaction plate (Applied Biosystems) and target gene expression was compared using the ΔΔCT method. Cycling and annealing temperatures were set according to the master mix manufacturer’s instructions. Data are represented relative to GAPDH.

### Immunocytochemistry and *in vitro* dendritic spine staining

FHC immunocytochemistry was performed as described previously (Pitcher et al., 2014), with minor modifications. Cells were washed with PBS, fixed with 2% paraformaldehyde (PFA) in PBS for 10 min at room temperature, followed by 4% PFA in PBS for 20 min at 4°C. Neurons were then permeabilized with 0.1% Triton X-100 in PBS for 5 min, and blocked with 5% normal goat serum (Jackson ImmunoResearch 005-000) in PBS for 30 min. Then, neurons were stained with antibodies against β-III Tubulin (Covance MMS-435P, RRID:AB_2313773, 1:1000) and FHC (Santa Cruz Biotechnology sc-25617, RRID:AB_2232020, 1:500) in blocking buffer overnight at 4°C. After three PBS washes, goat anti-mouse Alexa Fluor 568 (Invitrogen A11004, RRID:AB_2534072, 1:500), and goat anti-rabbit Alexa Flour 488 (Invitrogen A11008, RRID:AB_143165, 1:500) secondary antibodies in blocking buffer were added to the neurons for 1 h at room temperature. All coverslips were counterstained with Hoechst (Invitrogen 33342, 1:10,000) for 10 min before mounting. After staining, coverslips were rinsed in ultrapure water, mounted using VECTASHIELD mounting media (Vector Laboratories H-1000), sealed with nail polish, and stored at –20°C in the dark until use.

Dendritic spine studies *in vitro* used a modified approach. Neurons were cultured for 21 d, and then fixed as described above. Fixed neurons were permeabilized with 0.1% Triton X-100 for 5 min and blocked with 5% normal goat serum for 30 min. Then, the cells were stained with antibodies against MAP2 (Millipore AB5622, RRID:AB_91939, 1:1000) in blocking buffer overnight at 4°C, followed by a goat anti-rabbit Alexa Fluor 568 secondary antibody (Invitrogen A11011, RRID:AB_143157, 1:250) for 1 h. Cells were counterstained with Hoechst (Invitrogen 33342, 1:10,000) and Alexa Fluor 488 phalloidin (Invitrogen A12379, 25 μg/ml) for 15 min. After staining, coverslips were rinsed in ultrapure water, mounted with ProLong Gold Antifade Mountant (Invitrogen P36930), sealed with nail polish, and stored at –20°C in the dark until use. Neurons were imaged with an Olympus FLUOVIEW FV3000 confocal microscope.

### Animals

This study used male and female Holtzman rats (Harlan/Envigo) that were kept in Association for Assessment and Accreditation of Laboratory Animal Care-accredited University facilities in accordance with the National Institutes of Health guidelines and institutional approval by the Institutional Animal Care and Use Committee. As described previously (Sengupta et al., 2009; Shimizu et al., 2011a; Pitcher et al., 2014), E17 Holtzman rat embryos (or their P4 litters, either sex) were used as tissue sources for neuronal and glial cultures, respectively. Holtzman rat pups were also used for *in vivo* morphine treatments (Pitcher et al., 2014). PFC brain tissue from these rats was used for both immunohistochemistry and dendritic spine studies.

### *In vivo* morphine treatments and tissue collection

Male and female Holtzman rats were subcutaneously implanted with extended-release morphine pellets (NIH-NIDA, 25 mg), or vehicle pellets in the flank during the third postnatal week, as previously described (Pitcher et al., 2014). The rats were anesthetized with isoflurane and placed on a heating pad during surgical procedures. First, the implantation area was shaved, and betadine antiseptic (povidone-iodine) was applied to the skin before surgery. An incision through the skin was made with a sterile scalpel, and one 25-mg morphine pellet was subcutaneously implanted with sterile forceps. The incision was then closed with sterile wound clips (VWR 101326-476) and treated with a triple antibiotic ointment (neomycin, polymyxin B sulfates, and bacitracin zinc). The animals were placed on a heating pad in an empty cage until they recovered from anesthesia, and then placed back in their normal cages. Two days later, the incision was re-opened and the previous pellet was removed. The subcutaneous pocket was cleaned with sterile 0.9% saline, followed by implantation of two new 25-mg morphine or vehicle pellets. The incision was again closed with sterile wound clips and treated with triple antibiotic ointment. Two days after the second surgery (96-h total morphine exposure), brain tissue was collected for downstream analyses. For immunohistochemistry studies, rats were anesthetized with an intraperitoneal injection of ketamine (80 mg/kg; PennVet 50989-996-06)/xylazine (10 mg/kg; PennVet 50989-149-11) solution and perfused through the left ventricle with 50-ml warm 0.9% saline, followed by 200-ml room temperature 4% PFA in PBS. Brain tissue was immediately extracted and post-fixed in 4% PFA in PBS for 24 h at 4°C, then sent to our pathology core for paraffin embedding. For dendritic spine studies, rats were anesthetized with an intraperitoneal injection of ketamine (80 mg/kg)/xylazine (10 mg/kg) solution, followed by decapitation and rapid removal of the brain. Dissected brains were fixed in 4% PFA for 1 h, washed 3 times with PBS, and immediately sectioned at 150-μm thickness with a vibratome.

### Immunohistochemistry

Immunohistochemistry was performed as previously reported, with modifications (Pitcher et al., 2013). Paraffin blocks containing mPFC were sectioned at 5-µm thickness on a microtome. Sections were deparaffinized with xylene substitute (Thermo Scientific 9990505) for 30 min, followed by tissue rehydration in descending concentrations of ethanol for 5 min each (100% 2×, 95%, 90%, 70% 2×), and then distilled water. Antigen retrieval solution (Thermo Fisher 0050000) was prepared according to manufacturer’s instructions, and the solution was heated to 95°C in a water bath before adding tissue sections for 1 h. After the tissue cooled to room temperature, a 10% H_2_O_2_, 10% methanol (Fisher Scientific A412-4) in PBS solution was added to sections for 30 min to block endogenous peroxidases. Tissue was then blocked with 10% normal goat serum for 1 h at room temperature, followed by primary antibody incubation in a 2% normal goat serum solution overnight at 4°C. Tissue was stained with primary antibodies against NeuN (Cell Signaling Technology 24307, RRID:AB_2651140, 1:400), and FHC [H-53] (Santa Cruz Biotechnology sc-25617, RRID:AB_2232020, 1:100). After three PBS washes, sections were incubated in a Poly-HRP secondary antibody solution provided in the tyramide amplification kit (Thermo Fisher B40943) for 1 h. Tyramide reagent preparation and signal amplification were conducted according to the manufacturer’s instructions. In multiplex staining preparations, primary and secondary antibodies were stripped from the tissue by heat mediated antigen retrieval as described above. On completion of the staining protocol, slides were washed in distilled water, mounted with Prolong Gold Antifade Mountant (Invitrogen P36930), and sealed with nail polish. Slides were stored at room temperature in the dark until imaged.

### Multispectral imaging and analysis of IHC-stained brain tissue

Imaging and analysis was performed as previously described (Pitcher et al., 2013), with modifications. Briefly, fluorescent microscopy coupled with multispectral image analysis was used to identify individual neurons immunostained for NeuN (Cell Signaling Technology 24307, 1:400) within layer 2/3 of the mPFC prelimbic region. Within those neurons, the average fluorescent signal of FHC [H-53] immunostaining (Santa Cruz Biotechnology sc-25617, 1:100), a semi-quantitative measure of FHC expression, was measured. FHC average fluorescent signals were measured from at least 1000 layer 2/3 neurons in two separate slices from each animal, and these values were averaged to one value for each animal before statistical analysis. Immunohistochemical staining, image acquisition, and analysis were each performed by different people, and two people both blinded to the treatment condition separately performed analysis.

### DiOlistic staining of brain sections

Frontal cortex tissue was sectioned at 150-μm thickness with a vibratome. DiOlistic labeling was performed according to published techniques (Seabold et al., 2010). Tungsten beads (300 mg; Bio-Rad 1652269) were suspended in 99.5% pure methylene chloride (Fisher Scientific D37), and sonicated in a water bath for 1 h. Crystalized DiI (14.5 mg; Invitrogen D282) was dissolved in methylene chloride and protected from light. Following sonication, 100 μl of the tungsten bead solution was placed on a glass slide and 100 μl of DiI solution titrated on top, which was slowly mixed with a micropipette. The dried bead/dye mixture was scraped onto weighing paper with a razor blade, placed into a 15-ml conical tube with 3 mL distilled and deionized water, and sonicated in a water bath for 20 min. The bead/dye mixture was drawn into Tezfel tubing coated with polyvinylpyrrolidone (Fisher Scientific BP431-100), and dried with nitrogen gas for 1 h. Once dry, tubing was cut into 13 mm cartridges and loaded into the Helios Gene Gun (Bio-Rad). Helium gas flow was adjusted to 120 PSI and bullets were delivered to slices through 3 μm pore filter paper. Slices were quickly washed three times with PBS and stored overnight at 4°C to allow diffusion of the dye. The following day, slices were mounted using ProLong Gold Antifade Mountant (Invitrogen P36930), and stored at 4°C in the dark until imaging.

### Dendritic spine analysis *ex vivo* and *in vitro*

Dendrites in layer 2/3 pyramidal neurons from the prelimbic region of the mPFC, or dendrites from primary neuronal cultures were imaged with an Olympus FLUOVIEW FV3000 using a 100× objective at 0.15 μm per Z-step. Neurolucida 360 software was used to quantify dendritic spines, and classify them into their respective morphologies as previously reported (Rodriguez et al., 2008). For *ex vivo* studies, eight dendrites, at least 100–150 μm in length, were analyzed from eight separate neurons and averaged together as a single data point for each animal. For *in vitro* studies, four dendrites of at least 100–150 μm length from each coverslip were analyzed, and three coverslips were imaged for each condition per experiment. Each coverslip was averaged as a single data point and the experiment was repeated across three separate neuronal dissections. Different researchers were involved in treatments and imaging/analyses and investigators responsible for imaging and analyses were blinded to treatment.

### Intracellular levels of iron

Cortical neurons used for calcein assays were plated in black walled 96 well plates, at 20,000 neurons/well. Following drug treatments, calcein-AM (200 nM in HEPES buffered saline; Sigma 17783) was added to the cultures for 30 min, allowing for neuronal uptake and cleavage of the AM moiety, trapping the probe inside the neurons. Then, calcein-AM containing medium was removed and the cells were washed once with sterile HBS before imaging. Calcein fluorescence was measured in a fluorescence plate reader (Victor II) from PerkinElmer at 485-nm excitation and 535-nm emission (Cozzi et al., 2000). In cellular systems, calcein fluorescence selectively responds to altered iron levels (Epsztejn et al., 1997; Thomas et al., 1999) and is not sensitive to calcium and magnesium levels at cytoplasmic pH (Breuer et al., 1995).

Endolysosome iron level was measured using FeRhoNox-1 (Goryo Chemicals GC901), which selectively stains Fe^2+^ in endolysosomes and Golgi (Hirayama et al., 2013). For studies in cortical neurons, 12 DIV cells on coverslips were transfected with BacMam 2.0 LAMP1-GFP (Thermo Fisher C10507) according to the manufacturer’s instructions. Following transfection, neurons were loaded with FeRhoNox-1 (10 µM in HBSS; 1 h) at 37°C and washed with HBSS before treatments. Endolysosome iron measurements were achieved by measuring FeRhoNox-1 fluorescence at 537 nm excitation and 569 nm emission (Mukaide et al., 2014) in LAMP1-GFP-positive areas. Cytosolic iron level was measured with phen green SK, diacetate (Thermo Fisher P14313), which stains Fe^2+^ in the cytosol (Petrat et al., 2001). Similarly to calcein, phen green SK is not sensitive to cellular calcium and magnesium (Petrat et al., 1999; Reynolds, 2004). For studies in cortical neurons, 12 DIV cells on coverslips were transfected with BacMam 2.0 LAMP1-RFP (Thermo Fisher C10597) as before. Then, neurons were loaded with phen green SK (1 µM in PBS, 30 min) at 37°C, washed with PBS before treatments. Phen green fluorescence intensity was measured outside of LAMP1-RFP-positive areas by confocal microscopy using Alexa Fluor 488 settings (Rauen et al., 2003). As iron quenches phen green SK and calcein fluorescence, the inverse of fluorescence was plotted for these dyes to represent relative levels of cellular iron.

In studies of hippocampal neurons and U87MG cells, endolysosomes were identified by labeling cells with a combination of LysoTracker (Invitrogen L7528) and CellLight Golgi-GFP (Invitrogen C10592) according to the manufacturer’s instructions. Then, cells were loaded with FeRhoNox-1 as above, and FeRhoNox-1 fluorescence intensity was quantified in LysoTracker-positive areas. Likewise, these cells were labeled with LysoTracker and CellLight Golgi-RFP (Invitrogen C10593) for cytoplasmic iron measurements with phen green SK. See (Espósito et al., 2002) for a review of the iron sensors and chelators used in this study.

### Endolysosome pH analysis

Cortical neurons at 12 DIV were transfected with LAMP-1 GFP as above, and then loaded with 10 µg each of pH-sensitive pHrodo dextran (Thermo Fisher P10361) and pH-insensitive Alexa Fluor 647 dextran (Thermo Fisher D22914) overnight. The following morning, dextran containing medium was washed with PBS, and neurons incubated in fresh medium for 3 h. Following drug treatments, neurons were imaged by fluorescence microscopy. The ratio of 668/585 was measured and converted to pH using an intracellular pH calibration kit (Thermo Fisher Scientific, P35379) as described in (Johnson et al., 2016). Only LAMP1-GFP and dextran-positive endolysosomes were included in the analysis.

An alternative protocol was used for studies of endolysosome pH in U87MG cells. Cells were incubated with the ratiometric probe LysoSensor DND-160 (Invitrogen L7545; 1 µM) for 10 min, washed three times with PBS, and then analyzed with fluorescence microscopy at excitation wavelengths of 340 nm and 380 nm and an emission wavelength of 510 nm (Hui et al., 2012). Endolysosomes were differentiated from Golgi by adding CellLight Golgi-RFP (Invitrogen C10593; 2 µl/10 k cells) and incubating cells overnight at 37°C.

### Experimental design and statistical analysis

Cultures and animals were randomly assigned to groups. Numbers of animals per experimental group were determined by power analysis based on previous experiments and/or published data. Immunohistochemical staining, image acquisition, and analysis were each performed by different people, and two people both blinded to the treatment condition separately performed analysis. All biological and chemical reagents are from widely established commercial sources and further validated in house using positive and negative controls as feasible. Each experiment was performed at least three times; *in vitro* experiments used neurons derived from three independent litters, and *in vivo* experiments used four to six animals per treatment group, unless otherwise listed in figure legends. Data are reported as mean ± SEM. Statistical significance was determined using GraphPad Prism version 7.00 (RRID:SCR_002798) and was defined as *p* ≤ 0.05. Distribution of the data were assessed by the Shapiro–Wilk normality test. For normally distributed data comparing two groups, we used a two-tailed Student’s *t* test with a 5% significance level. For normally distributed data comparing more than two groups, we used a one-way ANOVA with a 5% significance level and Tukey’s or Dunnett’s multiple comparisons test. For spine morphology and dual FHC/FLC data, we used two-way ANOVA with Tukey’s multiple comparisons test for more than two groups, or Sidak’s multiple comparisons test for two groups. Individual statistical tests and multiple comparisons corrections used, and the results of these tests are listed in each figure legend and the statistics table (Table 1).

**Table 1.** Statistics table

**Figures**	**Data structure**	**Type of test**	**Statistical information**
Figure 1*A*	Normal distribution	One-way ANOVA	*F*_(6,14)_ = 52.697, *p* < 0.0001
Vehicle vs 0.01 μM		Dunnett's multiple comparisons test	CI: –1.8649 to –0.17292
Vehicle vs 0.1 μM		Dunnett's multiple comparisons test	CI: –3.6802 to –1.9882
Vehicle vs 1 μM		Dunnett's multiple comparisons test	CI: –3.9667 to –2.2748
Vehicle vs 10 μM		Dunnett's multiple comparisons test	CI: –3.5352 to –1.8432
Vehicle vs FAC		Dunnett's multiple comparisons test	CI: –4.198 to –2.5061
Vehicle vs DFO		Dunnett's multiple comparisons test	CI: –0.77749 to 0.91446
Figure 1*B*	Normal distribution	One-way ANOVA	*F*_(3,8)_ = 6.2933, *p* = 0.0168
Vehicle vs morphine		Dunnett's multiple comparisons test	CI: –1.9825 to –0.01226
Vehicle vs PTX		Dunnett's multiple comparisons test	CI: –1.0416 to 0.92856
Vehicle vs PTX + Mor		Dunnett's multiple comparisons test	CI: –0.53893 to 1.4313
Figure 1*C*	Normal distribution	One-way ANOVA	*F*_(7,16)_ = 94.711, *p* < 0.0001
Vehicle vs 30 m Mor		Dunnett's multiple comparisons test	CI: –0.49618 to 1.6318
Vehicle vs 6 h Mor		Dunnett's multiple comparisons test	CI: –1.2963 to 0.8317
Vehicle vs 24 h Mor		Dunnett's multiple comparisons test	CI: –1.6376 to 0.49035
Vehicle vs 24 h FAC		Dunnett's multiple comparisons test	CI: –6.7161 to –4.5881
Vehicle vs 1 h TNF		Dunnett's multiple comparisons test	CI: –2.109 to 0.019
Vehicle vs 3 h TNF		Dunnett's multiple comparisons test	CI: –6.4336 to –4.3057
Vehicle vs 24 h TNF		Dunnett's multiple comparisons test	CI: –0.98676 to 1.1412
Figure 1*D*	Normal distribution	One-way ANOVA	*F*_(3,42)_ = 0.38357, *p* = 0.7654
Vehicle vs 30 m Mor		Dunnett's multiple comparisons test	CI: –0.23702 to 0.11747
Vehicle vs 6 h Mor		Dunnett's multiple comparisons test	CI: –0.24894 to 0.10556
Vehicle vs 24 h Mor		Dunnett's multiple comparisons test	CI: –0.21279 to 0.14933
Figure 2*B*, cytoplasmic	Normal distribution	One-way ANOVA	*F*_(3,8)_ = 24.28, *p* = 0.0002
Vehicle vs 3 h Mor		Dunnett's multiple comparisons test	CI: –1.002 to 0.4371
Vehicle vs 6 h Mor		Dunnett's multiple comparisons test	CI: –1.629 to –0.1897
Vehicle vs 24 h Mor		Dunnett's multiple comparisons test	CI: –2.684 to –1.245
Figure 2*B*, nuclear	Normal distribution	One-way ANOVA	*F*_(3,8)_ = 1.644, *p* = 0.2549
Vehicle vs 3 h Mor		Dunnett's multiple comparisons test	CI: –0.6596 to 0.767
Vehicle vs 6 h Mor		Dunnett's multiple comparisons test	CI: –0.7541 to 0.6725
Vehicle vs 24 h Mor		Dunnett's multiple comparisons test	CI: –1.151 to 0.2751
Figure 3*A*, spine density	Normal distribution	Two-tailed, unpaired *t* test	*t*_(16)_ = 9.372, CI: –2.825 to –1.783
Figure 3*A*, spine morphology	Normal distribution	Two-way ANOVA	Interaction *F*_(3,64)_ = 13.9, *p* < 0.0001Treatment *F*_(3,64)_ = 151.9, *p* < 0.0001Morphology *F*_(1,64)_ = 81.83, *p* < 0.0001
Vehicle, morphine			
Thin		Sidak's multiple comparisons test	CI: 0.7779 to 1.428
Stubby		Sidak's multiple comparisons test	CI: 0.007506 to 0.6573
Mushroom		Sidak's multiple comparisons test	CI: 0.4853 to 1.135
Filopodia		Sidak's multiple comparisons test	CI: –0.2768 to 0.373
Figure 3*B*, spine density	Normal distribution	One-way ANOVA	*F*_(4,40)_ = 50.32, *p* < 0.0001
Vehicle vs 0.01 μM		Tukey's multiple comparisons test	CI: 0.0951 to 1.616
Vehicle vs 0.1 μM		Tukey's multiple comparisons test	CI: 1.048 to 2.569
Vehicle vs 1 μM		Tukey's multiple comparisons test	CI: 2.003 to 3.524
Vehicle vs 10 μM		Tukey's multiple comparisons test	CI: 2.487 to 4.008
0.01 vs 0.1 μM		Tukey's multiple comparisons test	CI: 0.1923 to 1.713
0.1 vs 1 μM		Tukey's multiple comparisons test	CI: 0.1951 to 1.716
1 vs 10 μM		Tukey's multiple comparisons test	CI: –0.2771 to 1.244
Figure 3*B*, spine morphology	Normal distribution	Two-way ANOVA	Interaction *F*_(12,160)_ = 21.58, *p* < 0.0001Morphology *F*_(3,160)_ = 956.9, *p* < 0.0001Treatment *F*_(4,160)_ = 42.9, *p* < 0.0001
Filopodia			
Vehicle vs 0.01 μM		Tukey's multiple comparisons test	CI: –0.4073 to 0.3795
Vehicle vs 0.1 μM		Tukey's multiple comparisons test	CI: –0.1906 to 0.5962
Vehicle vs 1 μM		Tukey's multiple comparisons test	CI: –0.3156 to 0.4712
Vehicle vs 10 μM		Tukey's multiple comparisons test	CI: –0.2767 to 0.5101
Mushroom			
Vehicle vs 0.01 μM		Tukey's multiple comparisons test	CI: –0.1934 to 0.5934
Vehicle vs 0.1 μM		Tukey's multiple comparisons test	CI: 0.006604 to 0.7934
Vehicle vs 1 μM		Tukey's multiple comparisons test	CI: 0.08438 to 0.8712
Vehicle vs 10 μM		Tukey's multiple comparisons test	CI: 0.1983 to 0.9851

Stubby			
Vehicle vs 0.01 μM		Tukey's multiple comparisons test	CI: –0.3934 to 0.3934
Vehicle vs 0.1 μM		Tukey's multiple comparisons test	CI: –0.4378 to 0.349
Vehicle vs 1 μM		Tukey's multiple comparisons test	CI: –0.3434 to 0.4434
Vehicle vs 10 μM		Tukey's multiple comparisons test	CI: –0.3295 to 0.4573
Thin			
Vehicle vs 0.01 μM		Tukey's multiple comparisons test	CI: 0.4372 to 1.224
Vehicle vs 0.1 μM		Tukey's multiple comparisons test	CI: 0.803 to 1.59
Vehicle vs 1 μM		Tukey's multiple comparisons test	CI: 1.77 to 2.557
Vehicle vs 10 μM		Tukey's multiple comparisons test	CI: 2.114 to 2.9
0.01 vs 0.1 μM		Tukey's multiple comparisons test	CI: –0.02756 to 0.7592
0.1 vs 1 μM		Tukey's multiple comparisons test	CI: 0.5741 to 1.361
1 vs 10 μM		Tukey's multiple comparisons test	CI: –0.05034 to 0.7365
Figure 3*C*, spine density	Normal distribution	One-way ANOVA	*F*_(5,42)_ = 15.29, *p* < 0.0001
Vehicle vs morphine		Tukey's multiple comparisons test	CI: 1.608 to 3.41
Vehicle vs PTX		Tukey's multiple comparisons test	CI: 0.0146 to 1.817
Vehicle vs PTX + Mor		Tukey's multiple comparisons test	CI: –0.3166 to 1.485
Vehicle vs CTAP		Tukey's multiple comparisons test	CI: 0.2709 to 2.073
Vehicle vs CTAP + Mor		Tukey's multiple comparisons test	CI: 0.09273 to 1.895
PTX vs PTX + Mor		Tukey's multiple comparisons test	CI: –1.232 to 0.5698
CTAP vs CTAP + Mor		Tukey's multiple comparisons test	CI: –1.079 to 0.7229
Figure 3*C*, spine morphology	Normal distribution	Two-way ANOVA	Interaction *F*_(15,168)_ = 9.193, *p* < 0.0001Morphology *F*_(3,168)_ = 1448, *p* < 0.0001Treatment *F*_(5,168)_ = 17.39, *p* < 0.0001
Filopodia			
Vehicle vs morphine		Tukey's multiple comparisons test	CI: –0.3425 to 0.4612
Vehicle vs PTX		Tukey's multiple comparisons test	CI: –0.3894 to 0.4144
Vehicle vs PTX + Mor		Tukey's multiple comparisons test	CI: –0.4519 to 0.3519
Vehicle vs CTAP		Tukey's multiple comparisons test	CI: –0.3675 to 0.4362
Vehicle vs CTAP + Mor		Tukey's multiple comparisons test	CI: –0.3487 to 0.455
Mushroom			
Vehicle vs morphine		Tukey's multiple comparisons test	CI: 0.1544 to 0.9581
Vehicle vs PTX		Tukey's multiple comparisons test	CI: –0.2019 to 0.6019
Vehicle vs PTX + Mor		Tukey's multiple comparisons test	CI: –0.2956 to 0.5081
Vehicle vs CTAP		Tukey's multiple comparisons test	CI: –0.08937 to 0.7144
Vehicle vs CTAP + Mor		Tukey's multiple comparisons test	CI: –0.1081 to 0.6956
PTX vs PTX + Mor		Tukey's multiple comparisons test	CI: –0.4956 to 0.3081
CTAP vs CTAP + Mor		Tukey's multiple comparisons test	CI: –0.4206 to 0.3831
Stubby			
Vehicle vs morphine		Tukey's multiple comparisons test	CI: –0.4644 to 0.3394
Vehicle vs PTX		Tukey's multiple comparisons test	CI: –0.5612 to 0.2425
Vehicle vs PTX + Mor		Tukey's multiple comparisons test	CI: –0.4206 to 0.3831
Vehicle vs CTAP		Tukey's multiple comparisons test	CI: –0.4144 to 0.3894
Vehicle vs CTAP + Mor		Tukey's multiple comparisons test	CI: –0.38 to 0.4237
Thin			
Vehicle vs morphine		Tukey's multiple comparisons test	CI: 1.404 to 2.208
Vehicle vs PTX		Tukey's multiple comparisons test	CI: 0.1481 to 0.9519
Vehicle vs PTX + Mor		Tukey's multiple comparisons test	CI: –0.03 to 0.7737
Vehicle vs CTAP		Tukey's multiple comparisons test	CI: 0.4544 to 1.258
Vehicle vs CTAP + Mor		Tukey's multiple comparisons test	CI: –0.03625 to 0.7675
PTX vs PTX + Mor		Tukey's multiple comparisons test	CI: –0.58 to 0.2237
CTAP vs CTAP + Mor		Tukey's multiple comparisons test	CI: –0.8925 to –0.08875
Figure 4*A*	Normal distribution	Two-tailed, unpaired *t* test	*t*_(6)_ = 2.717, CI: 0.6332 to 12.11
Figure 4*B*, spine density	Normal distribution	Two-tailed, unpaired *t* test	*t*_(10)_ = 8.482, CI: –7.026 to –4.103
Figure 4*B*, spine morphology	Normal distribution	Two-way ANOVA	Interaction *F*_(3,40)_ = 7.579, *p* = 0.0004Morphology *F*_(3,40)_ = 114, *p* < 0.0001Treatment *F*_(1,40)_ = 44.5, *p* < 0.0001
Vehicle, morphine			
Thin		Sidak's multiple comparisons test	CI: 1.922 to 4.195
Stubby		Sidak's multiple comparisons test	CI: –0.2348 to 2.038
Mushroom		Sidak's multiple comparisons test	CI: 0.4543 to 2.727

Filopodia		Sidak's multiple comparisons test	CI: –0.8738 to 1.399
Figure 5*A*	Normal distribution	Two-way ANOVA	Interaction *F*_(4,20)_ = 0.1919, *p* = 0.9398Treatment *F*_(4,20)_ = 12.94, *p* < 0.0001Expression *F*_(1,20)_ = 0.002895, *p* = 0.9576
Vehicle:FHC vs 30 m FAC:FHC		Tukey's multiple comparisons test	CI: –1.704 to 1.063
Vehicle:FHC vs 6 h FAC:FHC		Tukey's multiple comparisons test	CI: –1.8 to 0.9668
Vehicle:FHC vs 24 h FAC:FHC		Tukey's multiple comparisons test	CI: –2.94 to –0.1729
Vehicle:FHC vs DFO:FHC		Tukey's multiple comparisons test	CI: –1.299 to 1.468
Vehicle:FLC vs 30 m FAC:FLC		Tukey's multiple comparisons test	CI: –1.433 to 1.334
Vehicle:FLC vs 6 h FAC:FLC		Tukey's multiple comparisons test	CI: –1.762 to 1.005
Vehicle:FLC vs 24 h FAC:FLC		Tukey's multiple comparisons test	CI: –3.136 to –0.3684
Vehicle:FLC vs DFO:FLC		Tukey's multiple comparisons test	CI: –1.365 to 1.402
24 h FAC:FHC vs 24 h FAC:FLC		Tukey's multiple comparisons test	CI: –1.579 to 1.188
Figure 5*B*	Normal distribution	Two-way ANOVA	Interaction *F*_(3,24)_ = 1.244, *p* = 0.3157Treatment *F*_(3,24)_ = 22.94, *p* < 0.0001Expression *F*_(1,24)_ = 9.252, *p* = 0.0056
Vehicle:FHC vs 30 m Mor:FHC		Sidak's multiple comparisons test	CI: –1.581 to 0.5878
Vehicle:FHC vs 6 h Mor:FHC		Sidak's multiple comparisons test	CI: –2.202 to –0.03317
Vehicle:FHC vs 24 h Mor:FHC		Sidak's multiple comparisons test	CI: –3.17 to –1.001
Vehicle:FLC vs 30 m Mor:FLC		Sidak's multiple comparisons test	CI: –1.118 to 1.051
Vehicle:FLC vs 6 h Mor:FLC		Sidak's multiple comparisons test	CI: –1.596 to 0.5731
Vehicle:FLC vs 24 h Mor:FLC		Sidak's multiple comparisons test	CI: –2.356 to –0.1869
30 m Mor:FHC vs 30 m Mor:FLC		Sidak's multiple comparisons test	CI: –0.6212 to 1.548
6 h Mor:FHC vs 6 h Mor:FLC		Sidak's multiple comparisons test	CI: –0.4783 to 1.691
24 h Mor:FHC vs 24 h Mor:FLC		Sidak's multiple comparisons test	CI: –0.2706 to 1.899
Figure 5*C*	Normal distribution	Two-way ANOVA	Interaction *F*_(1,12)_ = 3.823, *p* = 0.0742Treatment *F*_(1,12)_ = 43.94, *p* < 0.0001Expression *F*_(1,12)_ = 3.814, *p* = 0.0745
Vehicle:FHC vs morphine:FHC		Tukey's multiple comparisons test	CI: –1.358 to –0.4659
Vehicle:FLC vs morphine:FHC		Tukey's multiple comparisons test	CI: –1.358 to –0.4657
Morphine:FHC vs morphine:FLC		Tukey's multiple comparisons test	CI: –0.03085 to 0.8613
Figure 6*B*	Normal distribution	One-way ANOVA	*F*_(4,8)_ = 112, *p* < 0.0001
Vehicle vs 0.1 μM		Dunnett's multiple comparisons test	CI: –1.472 to 2.722
Vehicle vs 1 μM		Dunnett's multiple comparisons test	CI: 1.293 to 5.487
Vehicle vs 10 μM		Dunnett's multiple comparisons test	CI: 8.804 to 13.49
Vehicle vs 100 μM		Dunnett's multiple comparisons test	CI: 10.07 to 14.76
Figure 6*D*	Normal distribution	One-way ANOVA	*F*_(4,13)_ = 47.98, *p* < 0.0001
Vehicle vs 0.1 μM		Dunnett's multiple comparisons test	CI: –26.25 to 4.68
Vehicle vs 1 μM		Dunnett's multiple comparisons test	CI: –34.28 to –6.624
Vehicle vs 10 μM		Dunnett's multiple comparisons test	CI: –60.4 to –35.66
Vehicle vs 100 μM		Dunnett's multiple comparisons test	CI: –61.63 to –36.89
Figure 6*E*, top	Normal distribution	One-way ANOVA	*F*_(3,8)_ = 180.2, *p* < 0.0001
Vehicle vs 0.1 μM		Dunnett's multiple comparisons test	CI: –0.2068 to 0.0702
Vehicle vs 1 μM		Dunnett's multiple comparisons test	CI: –0.6368 to –0.3598
Vehicle vs 10 μM		Dunnett's multiple comparisons test	CI: –1.127 to –0.8498
Figure 6*E*, bottom	Normal distribution	One-way ANOVA	*F*_(3,8)_ = 127.2, *p* < 0.0001
Vehicle vs morphine		Dunnett's multiple comparisons test	CI: –1.17 to –0.8066
Vehicle vs naloxone		Dunnett's multiple comparisons test	CI: –0.11 to 0.2534
Vehicle vs Nal + Mor		Dunnett's multiple comparisons test	CI: –0.21 to 0.1534
Figure 6*G*, EL	Normal distribution	Two-tailed, unpaired *t* test	*t*_(4)_ = 7.036, CI: –27.35 to –11.87
Figure 6*G*, cytosol	Normal distribution	Two-tailed, unpaired *t* test	*t*_(4)_ = 16.86, CI: 11.13 to 15.52
Figure 6*H*, EL	Normal distribution	One-way ANOVA	*F*_(3,8)_ = 541.2, *p* < 0.0001
Vehicle vs morphine		Tukey's multiple comparisons test	CI: 30.67 to 36.88
Vehicle vs naloxone		Tukey's multiple comparisons test	CI: –2.663 to 3.55
Vehicle vs Nal + Mor		Tukey's multiple comparisons test	CI: 4.1 to 10.31
Morphine vs Nal + Mor		Tukey's multiple comparisons test	CI: –29.68 to –23.46
Figure 6*H*, cytosol	Normal distribution	One-way ANOVA	*F*_(3,8)_ = 26.27, *p* = 0.0002
Vehicle vs morphine		Tukey's multiple comparisons test	CI: –14.9 to –5.351
Vehicle vs DFO		Tukey's multiple comparisons test	CI: –4.623 to 4.929
Vehicle vs DFO + Mor		Tukey's multiple comparisons test	CI: –3.169 to 6.383
Morphine vs DFO + Mor		Tukey's multiple comparisons test	CI: 6.957 to 16.51

Figure 6*I*	Normal distribution	One-way ANOVA	*F*_(3,8)_ = 45.05, *p* < 0.0001
Vehicle vs morphine		Tukey's multiple comparisons test	CI: –0.249 to –0.131
Vehicle vs naloxone		Tukey's multiple comparisons test	CI: –0.06895 to 0.04895
Vehicle vs Nal + Mor		Tukey's multiple comparisons test	CI: –0.119 to –0.001048
Morphine vs Nal + Mor		Tukey's multiple comparisons test	CI: 0.07105 to 0.189
Figure 7*A*	Normal distribution	One-way ANOVA	*F*_(7,16)_ = 87.91, *p* < 0.0001
Vehicle vs 30 m Mor		Dunnett's multiple comparisons test	CI: –678.2 to –19.94
Vehicle vs 3 h Mor		Dunnett's multiple comparisons test	CI: –1010 to –351.8
Vehicle vs 6 h Mor		Dunnett's multiple comparisons test	CI: –1033 to –374.9
Vehicle vs 24 h Mor		Dunnett's multiple comparisons test	CI: –741.5 to –83.21
Vehicle vs Phen		Dunnett's multiple comparisons test	CI: –27.91 to 630.4
Vehicle vs Phen + 24 h Mor		Dunnett's multiple comparisons test	CI: –208.3 to 449.9
Vehicle vs FAC		Dunnett's multiple comparisons test	CI: –2417 to –1759
Figure 7*B*	Normal distribution	One-way ANOVA	*F*_(5,14)_ = 13.72, *p* < 0.0001
Vehicle vs morphine		Dunnett's multiple comparisons test	CI: –0.954 to –0.06
Vehicle vs DFO		Dunnett's multiple comparisons test	CI: –0.06606 to 0.8279
Vehicle vs Mor + DFO		Dunnett's multiple comparisons test	CI: –0.009428 to 0.8182
Vehicle vs DTPA		Dunnett's multiple comparisons test	CI: –0.1879 to 0.7061
Vehicle vs Mor + DTPA		Dunnett's multiple comparisons test	CI: –0.9824 to –0.08839
Figure 7*C*, spine density	Normal distribution	One-way ANOVA	*F*_(6,56)_ = 24.21, *p* < 0.0001
Vehicle vs morphine		Tukey's multiple comparisons test	CI: 1.869 to 3.642
Vehicle vs FAC		Tukey's multiple comparisons test	CI: 1.494 to 3.267
Vehicle vs DFO		Tukey's multiple comparisons test	CI: 0.1525 to 1.925
Vehicle vs DFO + Mor		Tukey's multiple comparisons test	CI: 0.1108 to 1.884
Vehicle vs DTPA		Tukey's multiple comparisons test	CI: –0.3058 to 1.467
Vehicle vs DTPA + Mor		Tukey's multiple comparisons test	CI: 1.13 to 2.903
Morphine vs FAC		Tukey's multiple comparisons test	CI: –1.261 to 0.5114
DFO vs DFO + Mor		Tukey's multiple comparisons test	CI: –0.928 to 0.8447
DTPA vs DTPA + Mor		Tukey's multiple comparisons test	CI: 0.5497 to 2.322
Figure 7*C*, spine morphology	Normal distribution	Two-way ANOVA	Interaction *F*_(18,224)_ = 17.58Morphology *F*_(3,224)_ = 1991, *p* < 0.0001Treatment *F*_(6,224)_ = 31.61, *p* < 0.0001
Filopodia			
Vehicle vs morphine		Tukey's multiple comparisons test	CI: –0.254 to 0.504
Vehicle vs FAC		Tukey's multiple comparisons test	CI: –0.2623 to 0.4956
Vehicle vs DFO		Tukey's multiple comparisons test	CI: –0.2984 to 0.4595
Vehicle vs DFO + Mor		Tukey's multiple comparisons test	CI: –0.3355 to 0.4225
Vehicle vs DTPA		Tukey's multiple comparisons test	CI: –0.2568 to 0.5012
Vehicle vs DTPA + Mor		Tukey's multiple comparisons test	CI: –0.2512 to 0.5068
Mushroom			
Vehicle vs morphine		Tukey's multiple comparisons test	CI: 0.1044 to 0.8623
Vehicle vs FAC		Tukey's multiple comparisons test	CI: 0.07103 to 0.829
Vehicle vs DFO		Tukey's multiple comparisons test	CI: –0.1206 to 0.6373
Vehicle vs DFO + Mor		Tukey's multiple comparisons test	CI: –0.05953 to 0.6984
Vehicle vs DTPA		Tukey's multiple comparisons test	CI: –0.2068 to 0.5512
Vehicle vs DTPA + Mor		Tukey's multiple comparisons test	CI: 0.03769 to 0.7956
Morphine vs FAC		Tukey's multiple comparisons test	CI: –0.4123 to 0.3456
DFO vs DFO + Mor		Tukey's multiple comparisons test	CI: –0.3179 to 0.4401
DTPA vs DTPA + Mor		Tukey's multiple comparisons test	CI: –0.1345 to 0.6234
Stubby			
Vehicle vs morphine		Tukey's multiple comparisons test	CI: –0.3845 to 0.3734
Vehicle vs FAC		Tukey's multiple comparisons test	CI: –0.3706 to 0.3873
Vehicle vs DFO		Tukey's multiple comparisons test	CI: –0.4429 to 0.3151
Vehicle vs DFO + Mor		Tukey's multiple comparisons test	CI: –0.3956 to 0.3623
Vehicle vs DTPA		Tukey's multiple comparisons test	CI: –0.3956 to 0.3623
Vehicle vs DTPA + Mor		Tukey's multiple comparisons test	CI: –0.3929 to 0.3651
Thin			
Vehicle vs morphine		Tukey's multiple comparisons test	CI: 1.788 to 2.546
Vehicle vs FAC		Tukey's multiple comparisons test	CI: 1.457 to 2.215
Vehicle vs DFO		Tukey's multiple comparisons test	CI: 0.4081 to 1.166
Vehicle vs DFO + Mor		Tukey's multiple comparisons test	CI: 0.3432 to 1.101
Vehicle vs DTPA		Tukey's multiple comparisons test	CI: –0.07897 to 0.679

Vehicle vs DTPA + Mor		Tukey's multiple comparisons test	CI: 1.121 to 1.879
Morphine vs FAC		Tukey's multiple comparisons test	CI: –0.7095 to 0.04842
DFO vs DFO + Mor		Tukey's multiple comparisons test	CI: –0.4438 to 0.3142
DTPA vs DTPA + Mor		Tukey's multiple comparisons test	CI: 0.821 to 1.579

## Results

### Morphine post-transcriptionally upregulates FHC protein in the neuronal cytoplasm

**Figure 1.**
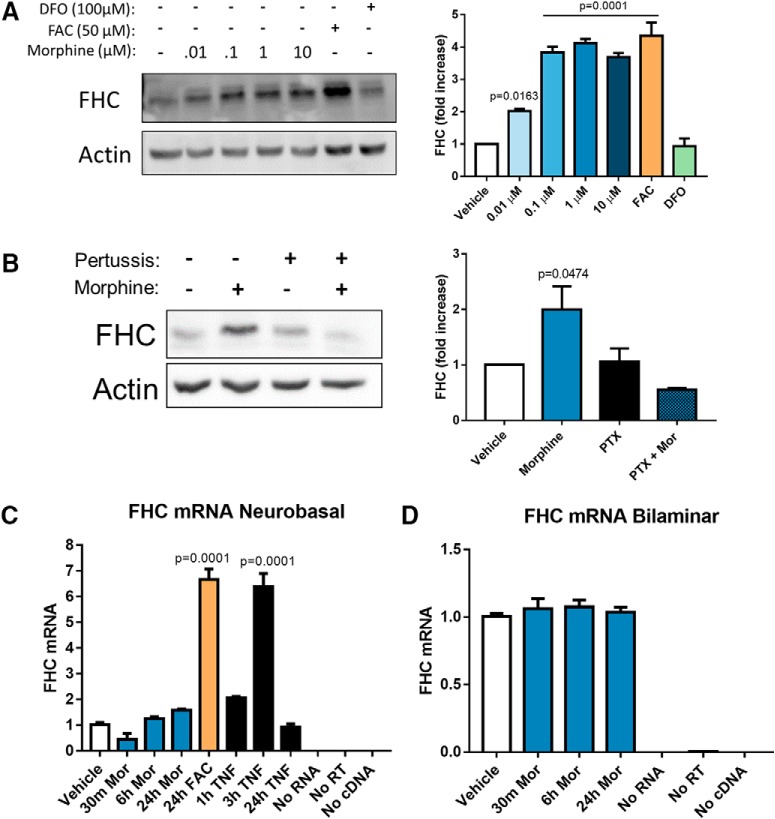
Morphine upregulates FHC protein without altering transcript levels. ***A***, Morphine dose dependently upregulates neuronal FHC. Neurobasal cultures were treated with morphine (0.01, 0.1, 1, or 10 µM) or vehicle for 24 h. Morphine significantly increased FHC protein level at every dose, and 1 µM produced a peak effect. Positive control cultures were iron-loaded with FAC (50 µM, 24 h), and negative control cultures were iron-chelated with DFO (100 µM, 24 h). Iron loading significantly increased FHC protein levels, while iron chelation did not alter FHC protein levels, showing that neurobasal cultures could predictably respond to altered iron levels through FHC synthesis; *F*_(6,14)_ = 52.697, *p* < 0.0001. ***B***, Blocking Gαi signaling inhibits morphine-mediated FHC upregulation in bilaminar cultures. Cultures were pre-treated with PTX (200 ng/ml) or vehicle for 2 h, followed by addition of morphine (1 µM, 24 h). Morphine alone significantly increased FHC protein levels, but pre-treatment with PTX completely blocked FHC upregulation by morphine; *F*_(3,8)_ = 6.2933, *p* = 0.0168. ***C***, Morphine does not change FHC transcript expression in neurobasal cultures. Cultures were treated with morphine (1 µM) for 30 min, 6 h, or 24 h before collection of total RNA. Morphine had no effect on FHC transcript expression as assessed by qPCR. Positive control cultures either iron loaded with a high concentration of FAC (100 µM) for 24 h or treated with TNFα (10 ng/ml) for 3 h significantly upregulated FHC transcripts, showing that the cultures were capable of increasing FHC gene expression; *F*_(7,16)_ = 94.711, *p* < 0.0001. ***D***, Morphine does not change FHC transcript expression in bilaminar cultures. As before, cultures were treated with morphine (1 µM) for 30 min, 6 h, or 24 h before collection of total RNA. Again, morphine had no effect on neuronal FHC transcript levels, even in the presence of a glial feeder layer; *N* = 4 experiments, *F*_(3,42)_ = 0.38357, *p* = 0.7654. In both ***C***, ***D***, FHC transcripts were quantified using the ΔΔCT method, and data are presented relative to GAPDH. All experiments analyzed by one-way ANOVA and Dunnett *post hoc*.

As a first step to understand the mechanisms of FHC modulation by µ-opioid agonists, we investigated whether morphine was able to upregulate FHC in a dose-dependent manner. We added 0.1–10 µM morphine to pure cortical neuronal cultures (neurobasal), lysed the neurons 24 h later, and measured FHC protein expression by Western blotting. Indeed, FHC protein levels increased in a dose-dependent manner, and 1 µM morphine produced a peak effect (Fig. 1*A*). Since FHC is upregulated by iron in many cell types (Torti and Torti, 2002), we iron-loaded positive control cultures with FAC (50 µM, 24 h) and iron-chelated negative control cultures with DFO (100 µM, 24 h). As expected, iron loading increased FHC expression and chelation did not (Fig. 1*A*), demonstrating that iron controls neuronal FHC protein levels as previously reported in non-neuronal cells (Torti and Torti, 2002).

Morphine-mediated FHC upregulation in cortical neurons depends on µOR activation, as this pathway is blocked by pre-treatment with the µOR antagonist CTAP (Sengupta et al., 2009) and altered in µOR deficient mice (Burbassi et al., 2010). µORs can signal through G-protein and β-arrestin pathways, which mediate distinct treatment and side effects (Manglik et al., 2016; Schmid et al., 2017). Since the Gαi-protein pathway is necessary for producing clinically useful analgesic effects of opioids (Tseng and Collins, 1996; Bohn et al., 1999; Soergel et al., 2014), we determined whether the Gαi-protein pathway is also involved in FHC upregulation. We blocked Gαi signaling in neuronal/glial cocultures (bilaminar) by pre-treatment with 200 ng/ml PTX, and then added morphine or vehicle to the PTX-containing medium. This experiment used bilaminar cultures to determine if the presence of glia altered neuronal FHC upregulation by morphine. At 24 h post-morphine treatment, we analyzed neuronal lysates for FHC expression by Western blotting. Morphine-mediated FHC upregulation was completely blocked by PTX (Fig. 1*B*), demonstrating that this pathway requires Gαi protein signaling, and suggesting that the glial feeder layer does not alter morphine’s ability to upregulate neuronal FHC. The necessity of µOR and Gαi-protein activation for FHC upregulation suggests that the majority of clinical and illicit opioid ligands, which also signal through these pathways (Sanchez-Blazquez et al., 2001), will upregulate neuronal FHC. However, FHC upregulation may still be affected by µOR β-arrestin signaling in a different capacity.

Next, we examined FHC gene expression in morphine-treated neurobasal and bilaminar cultures. We collected total neuronal RNA from 30 min to 24 h after morphine treatment and determined FHC transcript expression by quantitative RT-PCR. FHC transcript levels did not change following morphine treatment in both cultures (Fig. 1*C*,*D*), suggesting that morphine post-transcriptionally upregulated FHC protein. Positive control neurobasal cultures demonstrated that FHC transcripts could be increased by different stimuli, including TNFα (10 ng/ml; 3 h) and oxidative stress from massive iron loading with FAC (100 µM, 24 h; Fig. 1*C*).

FHC is translocated from the cytoplasm to the nucleus of neuronal and non-neuronal cells, suggesting that it has functions in both compartments (Cheepsunthorn et al., 1998; Surguladze et al., 2005; Li et al., 2006). Cytoplasmic FHC is involved in cellular iron storage and regulation of CXCR4 signaling at or near the cell membrane, since this is where the majority of neuronal CXCR4 is expressed (Shimizu et al., 2011b). Nuclear FHC protects DNA from oxidative damage and may control gene expression (Surguladze et al., 2004; Storr et al., 2009; Alkhateeb and Connor, 2010), suggesting that insufficient nuclear FHC levels may compromise DNA integrity. Since FHC localization underlies homeostatic processes as well as CXCR4 inhibition, we determined whether morphine also affected FHC localization.

**Figure 2.**
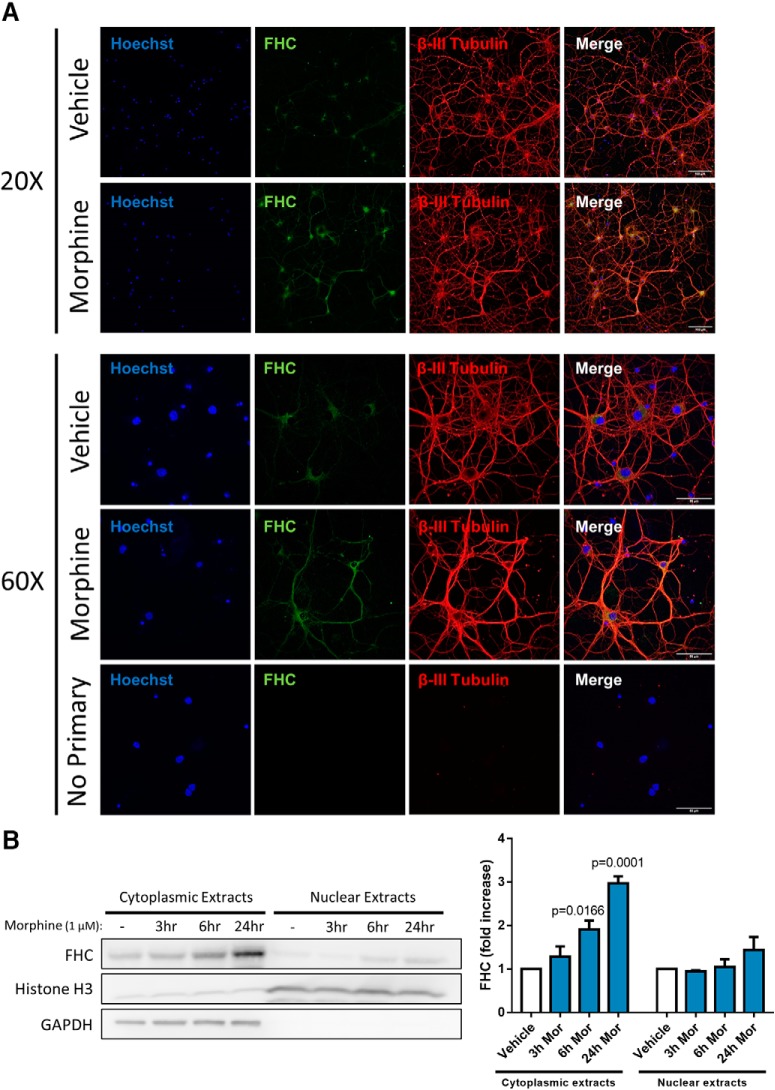
Morphine upregulates FHC protein in the neuronal cytoplasm. ***A***, FHC is expressed in the soma and processes of morphine-treated neurons. Neurobasal cultures were treated with morphine (1 µM, 24 h) or vehicle before fixation and immunostaining. Cultures were immunostained for FHC (green) and the neuronal marker β-III Tubulin (red), and counterstained with the nuclear marker Hoechst (blue). Images were acquired with 20× and 60× objectives. Morphine treatment visibly increased FHC staining in the soma and processes. One group of neurons was immunostained without both primary antibodies, showing that non-specific staining was negligible. ***B***, Morphine upregulates FHC in cytoplasmic extracts of neurobasal cultures. Cultures were treated with morphine (1 µM, 3, 6, or 24 h) or vehicle, and separated into cytosolic and nuclear extracts. Morphine dose dependently increased FHC protein levels in cytoplasmic extracts, and 6-h and 24-h treatments reached significance; *F*_(3,8)_ = 24.28, *p* = 0.0002. Conversely, morphine did not significantly alter FHC expression in nuclear extracts at any time; *F*_(3,8)_ = 1.644, *p* = 0.2549. Both experiments were analyzed by one-way ANOVA and Dunnett *post hoc*.

We analyzed FHC subcellular localization and expression in neurobasal cultures with confocal imaging and cellular fractionation approaches, respectively. In confocal imaging studies, we treated neurons with vehicle or morphine (1 µM, 24 h), immunostained for FHC (green) and the neuronal marker β-III Tubulin (red), and counterstained with Hoechst (blue) to visualize nuclei. Vehicle-treated neurons mainly displayed low-level FHC staining around the soma, while morphine-treated neurons displayed increased FHC staining in the soma and processes (Fig. 2*A*). In cellular fractionation studies, we treated neurons with vehicle or 1 µM morphine, lysed the cultures 3, 6, or 24 h later, and separated lysates into cytosolic and nuclear extracts to be analyzed by Western blotting. Morphine time dependently upregulated FHC in the cytoplasmic extracts but did not significantly alter FHC levels in the nuclear extracts (Fig. 2*B*). Together, these results show that morphine-mediated FHC upregulation occurs through mRNA translation in the cytoplasmic compartment, and that morphine does not significantly alter nuclear translocation of FHC.

### Morphine reduces the density of mature dendritic spine types *in vitr*o and *in viv*o

**Figure 3.**
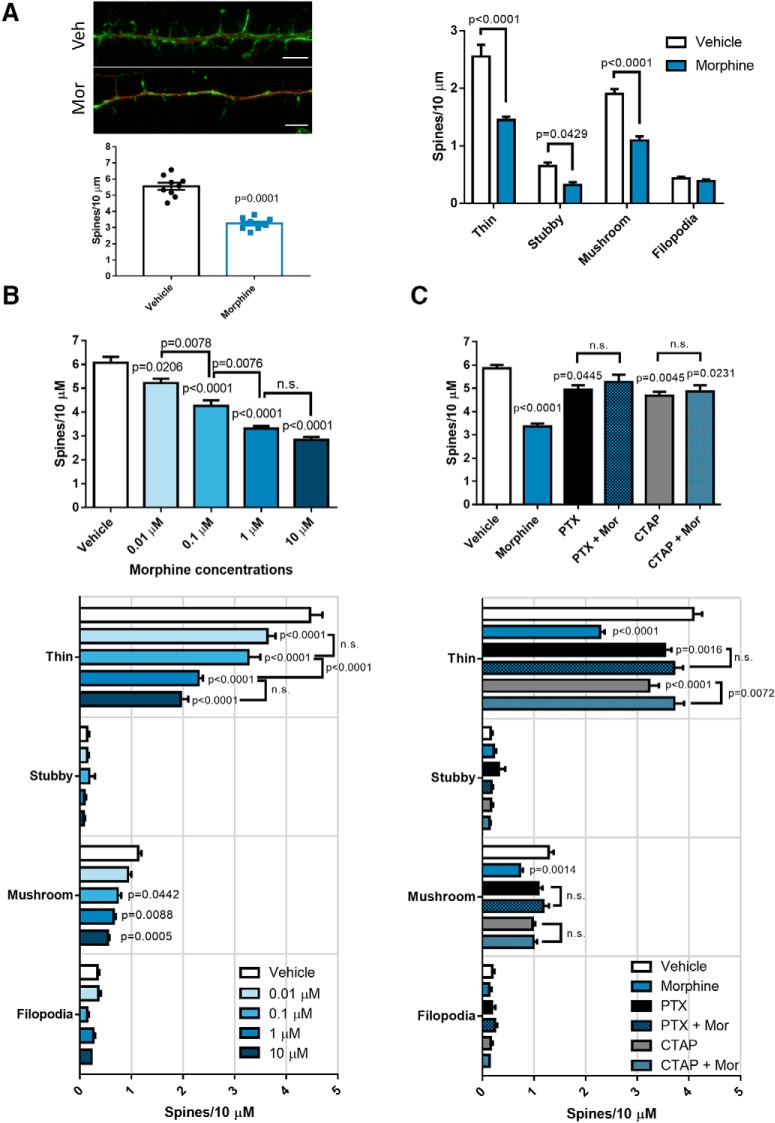
Morphine dose dependently reduces dendritic spine density and mature spine types through µOR and Gαi signaling. ***A***, Morphine reduced several dendritic spine types in neurobasal cultures. Cultures (20 DIV) were treated with morphine (1 µM, 24 h), followed by fixation and staining with antibodies against MAP2 and with phalloidin 488 counterstain to visualize dendritic spines in MAP2-positive neurons; scale bar = 5 µm. Morphine significantly reduced overall dendritic spine density (*t*_(16)_ = 9.372) and specifically reduced the density of thin, stubby, and mushroom spines. Dendritic spine density data were analyzed by two-tailed Student’s *t* test, while dendritic spine morphology data were analyzed by two-way ANOVA with Sidak’s multiple comparisons test (treatment *F*_(3,64)_ = 151.9, *p* < 0.0001; morphology *F*_(1,64)_ = 81.83, *p* < 0.0001). ***B***, Morphine decreases dendritic spine density in a dose-dependent manner. Neurobasal cultures (20 DIV) were treated with morphine (0.01, 0.1, 1, or 10 µM) or vehicle for 24 h before fixation. As in ***A***, treated cultures were stained with antibodies against MAP2, and counterstained with phalloidin 488 to visualize dendritic spines in MAP2-positive neurons. Morphine reduced overall dendritic spine density dose dependently, and each dose up to 1 µM reduced spine density significantly more than the previous dose; *F*_(4,40)_ = 50.32, *p* < 0.0001. Spine morphology analysis showed the same dose-dependent reduction of thin and mushroom spines. All morphine doses significantly reduced thin spine density, while only 0.1, 1, and 10 µM morphine significantly reduced mushroom spine density; treatment *F*_(4,160)_ = 42.9, *p* < 0.0001; spine morphology *F*_(3,160)_ = 956.9, *p* < 0.0001. ***C***, Morphine’s actions on dendritic spines depend on µOR and Gαi protein activation. Neurobasal cultures (20 DIV) were either treated with morphine (1 µM, 24 h) alone or pre-treated with the µOR antagonist CTAP (1 µM) or the Gαi protein inhibitor PTX (200 ng/ml) for 30 min/2 h before morphine treatment, respectively. Morphine alone significantly reduced dendritic spine density, which was blocked by cotreatment with both CTAP and PTX; *F*_(5,42)_ = 15.29, *p* < 0.0001. Spine morphology analysis revealed a similar pattern where morphine significantly reduced thin and mushroom spine density, which was rescued by PTX and CTAP pre-treatment; treatment *F*_(5,168)_ = 17.39, *p* < 0.0001; spine morphology *F*_(3,168)_ = 1448, *p* < 0.0001. *N* = 3 experiments for all panels. Spine density data were analyzed by one-way ANOVA and Tukey *post hoc*, while spine morphology data were analyzed by two-way ANOVA and Tukey *post hoc*.

To better characterize morphine’s effect on dendritic spines and its relationship with FHC, we examined whether morphine reduced specific types of spines, and whether spine reduction aligned with FHC levels. As a start, we treated neurobasal cultures (21 DIV) with vehicle or 1 µM morphine 24 h before fixation. Then we immunostained for the neuronal marker MAP2 (red), visualized dendritic spines by counterstaining with the actin-labeling probe phalloidin 488 (green), and analyzed total spine density and morphology with the Neurolucida 360 image analysis system. As expected, morphine treatment significantly reduced overall dendritic spine density, and an initial spine morphology analysis showed that morphine mainly reduced mushroom and thin spines with a modest reduction of stubby spines (Fig. 3*A*), suggesting that morphine-mediated FHC upregulation mostly reduces mature types of dendritic spines.

Next, we expanded our analysis of morphine’s effects on dendritic spine density and morphology *in vitro*. In a new group of cortical neurons treated with increasing concentrations of morphine (0.01, 0.1, 1, or 10 µM, 24 h) we saw that morphine reduced overall dendritic spine density dose dependently, and each dose up to 1 µM reduced spine density significantly more than the previous dose (Fig. 3*B*). Spine morphology analyses showed a similar dose-dependent spine reduction, as every dose of morphine reduced thin spine density, and 0.1–10 µM doses significantly reduced mushroom spine density (Fig. 3*B*). Since we have shown that morphine’s actions on dendritic spines required FHC upregulation (Pitcher et al., 2014), we next determined whether µOR activation and Gαi signaling, which are upstream of FHC upregulation, are also required. As before, we treated neuronal cultures with 1 µM morphine alone, or pre-treated with either the µOR antagonist CTAP (1 µM, 30 min pre-treatment), or the Gαi-protein inhibitor PTX (200 ng/ml; 2 h pre-treatment) before morphine treatment. In line with previous findings, morphine treatment alone significantly reduced overall dendritic spine density, which was prevented by pre-treatment with either CTAP or PTX (Fig. 3*C*). These results are mirrored in dendritic spine morphology analyses from the same neurons, where morphine alone significantly reduced thin and mushroom spines, and pre-treatment with CTAP or PTX blocked morphine’s effects (Fig. 3*C*). Stubby spine density did not change in these studies, further suggesting that morphine affects mature spine types. These experiments show that morphine-mediated dendritic spine reduction in cortical neurons requires µOR Gαi-protein signaling.

**Figure 4.**
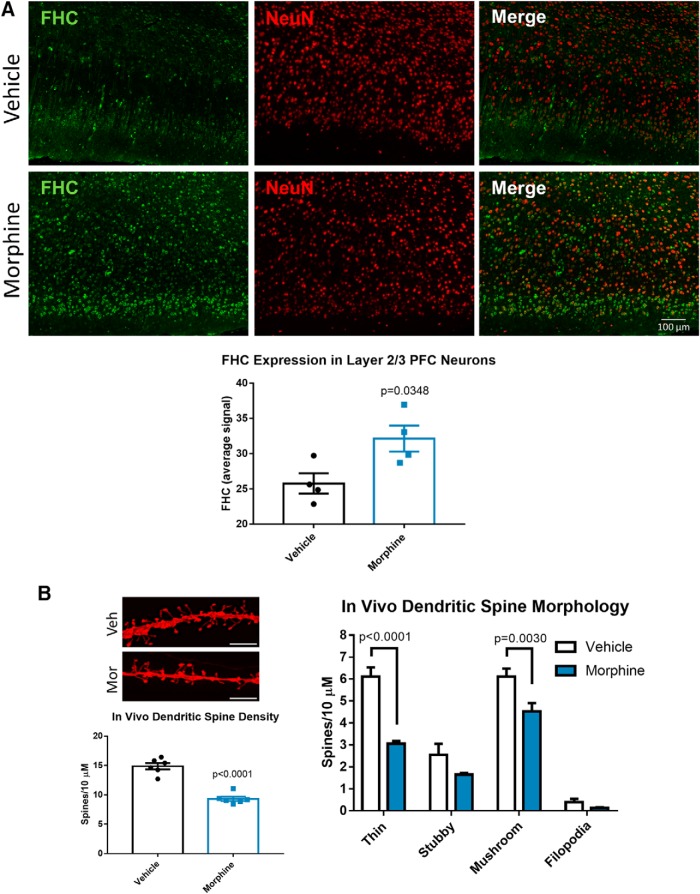
Morphine upregulates FHC and decreases mature dendritic spines in layer 2/3 neurons of the rat medial prefrontal cortex. ***A***, Morphine upregulates FHC in cortical neurons *in vivo*. Three-week-old Holtzman rats were treated with extended-release morphine pellets (25 mg) or placebo for 96 h as detailed in the methods, followed by perfusion and brain tissue collection. Brain sections were stained with antibodies against FHC (green) and the neuronal marker NeuN (red), and images were acquired with a 20× objective. Images were analyzed by measuring the staining intensity of FHC in NeuN-positive areas of the layer 2/3 prelimbic cortex of the mPFC. FHC staining intensity values from individual neurons were averaged to one value per rat, represented as one dot in the graph. FHC staining was significantly higher in neurons of morphine-treated rats; *N* = 4 rats per treatment group. Data analyzed by Student’s *t* test; *t*_(6)_ = 2.717. ***B***, Morphine reduced thin and mushroom dendritic spine density in PFC neurons. A different group of three-week-old Holtzman rats treated with morphine or placebo pellets were used for dendritic spine analysis. PFC-containing tissue slices were stained with DiI to visualize dendritic spines, as shown in the micrograph; scale bar = 5 µm. Morphine decreased the overall spine density of layer 2/3 prelimbic cortex neurons (*t*_(10)_ = 8.482), and specifically reduced the density of thin and mushroom spines. Stubby spines and filopodia were not significantly changed by morphine; *N* = 6 rats per treatment group. Spine density data were analyzed by Student’s *t* test, and morphology data were analyzed by two-way ANOVA with Sidak’s multiple comparisons test (treatment *F*_(1,40)_ = 44.5, *p* < 0.0001; morphology *F*_(3,40)_ = 114, *p* < 0.0001).

The following set of experiments focused on morphine’s effects *in vivo*. We subcutaneously implanted extended-release morphine pellets in the flank of Holtzman rats, ensuring continuous exposure to morphine for the treatment duration. Then we examined dendritic spines and FHC expression in the medial PFC because this brain area mediates learning and memory processes that are often disrupted in HAND (Dumitriu et al., 2010; Bloss et al., 2011; Morrison and Baxter, 2012). To visualize FHC expression in individual layer 2/3 mPFC neurons, we stained PFC brain sections for FHC (green) and the neuronal marker NeuN (red). Then we quantified FHC staining intensity in NeuN-positive neurons of the layer 2/3 medial PFC prelimbic region using the Nuance multispectral imaging system. For quantification and statistical analysis, we averaged all FHC staining intensity measurements from individual neurons to one value per animal. FHC was upregulated in medial PFC neurons from morphine-treated rats (Fig. 4*A*), which is in line with our previous studies in humans and macaques (Pitcher et al., 2014).

We used a second group of morphine-pellet-treated rats to study dendritic spine density and morphology changes of the layer 2/3 medial PFC prelimbic region. In this case, we stained PFC brain sections with DiI, a fluorescent lipophilic dye that labels dendritic spines, and then analyzed dendritic spine density and morphology with the Neurolucida 360 system. As expected, morphine treatment decreased total dendritic spine density, and spine morphology analysis showed that morphine reduced mature mushroom and thin spines but had no effect on immature filopodia and stubby spines (Fig. 4*B*). These experiments show that primary cortical neurons, as well as layer 2/3 medial PFC neurons are susceptible to opioid-induced FHC upregulation and synaptic injury targeting mature types of dendritic spines.

### Morphine-mediated efflux of endolysosomal iron drives FHC upregulation and reduction of mature types of dendritic spines

**Figure 5.**
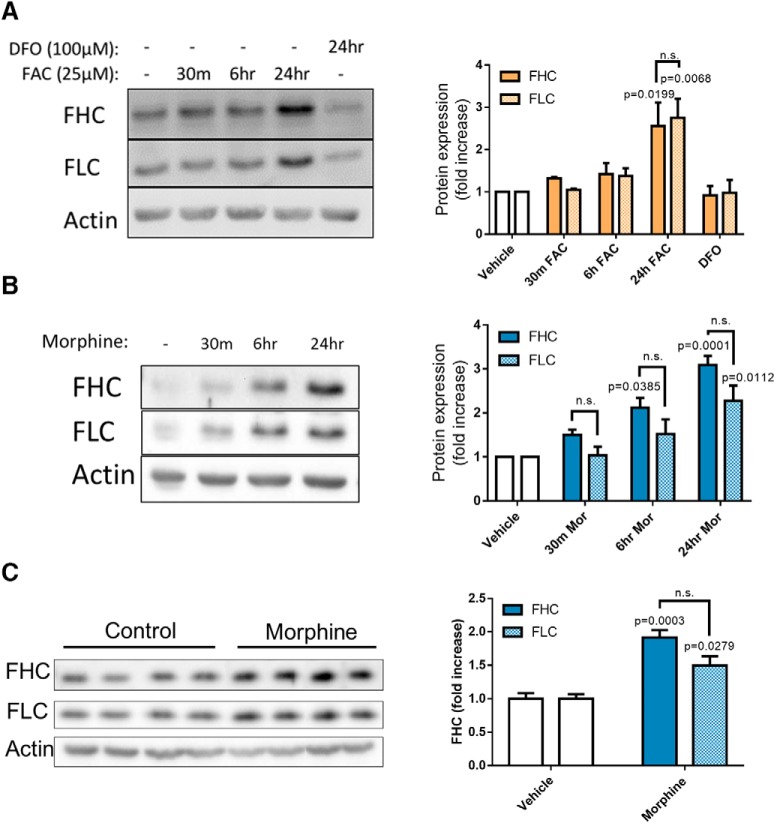
Morphine and iron upregulate FHC and FLC in cortical neurons. ***A***, Iron-loading upregulates FHC and FLC in neurobasal cultures. Cultures were iron-loaded with FAC (25 µM) for 30 min, 6 h, or 24 h before lysis. Additionally, a negative control culture was iron-chelated with DFO (100 µM, 24 h) before lysis. Iron loading with FAC significantly increased FHC and FLC but only after 24 h. FHC and FLC expression were not significantly different at any time after treatment; *N* = 3 experiments; treatment *F*_(4,20)_ = 12.94, *p* < 0.0001; FHC/FLC expression *F*_(1,20)_ = 0.0029, *p* = 0.9576. ***B***, Morphine upregulates FHC and FLC in neurobasal cultures. Cultures were treated with morphine (1 µM) or vehicle and lysed 30 min, 6 h, or 24 h after treatment. Morphine upregulated both FHC and FLC, but FHC was significantly upregulated at 6 h, while FLC reached significance at 24 h. However, the overall expression of FHC was not significantly different from FLC at each time point; *N* = 4 experiments; treatment *F*_(3,24)_ = 22.94, *p* < 0.0001; FHC/FLC expression *F*_(1,24)_ = 9.252, *p* = 0.0056. ***C***, Morphine-treated rats upregulate FHC and FLC in frontal cortex tissue. Three-week-old Holtzman rats were treated with extended-release morphine or placebo pellets for 96 h as described in Figure 4 and the Materials and Methods. After the treatment, rats were killed and frontal cortex tissue was dissected, homogenized, and analyzed by Western blotting. Morphine significantly increased FHC and FLC expression *in vivo*, similarly to the *in vitro* experiment in panel ***B***; *N* = 4 rats per treatment group; each column contains a homogenate from a different rat; treatment *F*_(1,12)_ = 43.94, *p* < 0.0001; FHC/FLC expression *F*_(1,12)_ = 3.814, *p* = 0.0745. All data were analyzed by two-way ANOVA and Tukey *post hoc*.

We suspected that morphine might control neuronal FHC expression by altering intracellular labile iron levels because FHC is involved in iron storage and oxidation, and its expression can be post-transcriptionally increased by iron (Hentze et al., 1987; Leibold and Munro, 1988; Hentze and Kühn, 1996). Iron also post-transcriptionally regulates ferritin light chain (FLC; Gray and Hentze, 1994; Hentze and Kühn, 1996), which self assembles with FHC to form the iron storing 24mer ferritin (Torti and Torti, 2002). Based on this literature, we first determined whether iron upregulated FHC and FLC in neurobasal cultures. Indeed, cultures iron loaded with 25 µM FAC for 24 h significantly upregulated FHC and FLC (Fig. 5*A*). Iron chelation with DFO (100 µM, 24 h) did not significantly change basal FHC or FLC expression (Fig. 5*A*). Next, we determined whether morphine also increased FHC and FLC expression in these cultures. Interestingly, morphine (1 µM) upregulated both FHC and FLC, and FHC levels increased faster than iron loading (Fig. 5*B*), suggesting that morphine may mobilize iron from intracellular stores. We also examined FHC and FLC protein expression in frontal cortex homogenates of rats implanted with morphine pellets by Western blotting and found a similar pattern of FHC and FLC upregulation compared to morphine-treated neuronal cultures (Fig. 5*C*). These data suggest that increased cellular iron is involved in morphine-mediated FHC/FLC upregulation *in vitro* and *in vivo*.

**Figure 6.**
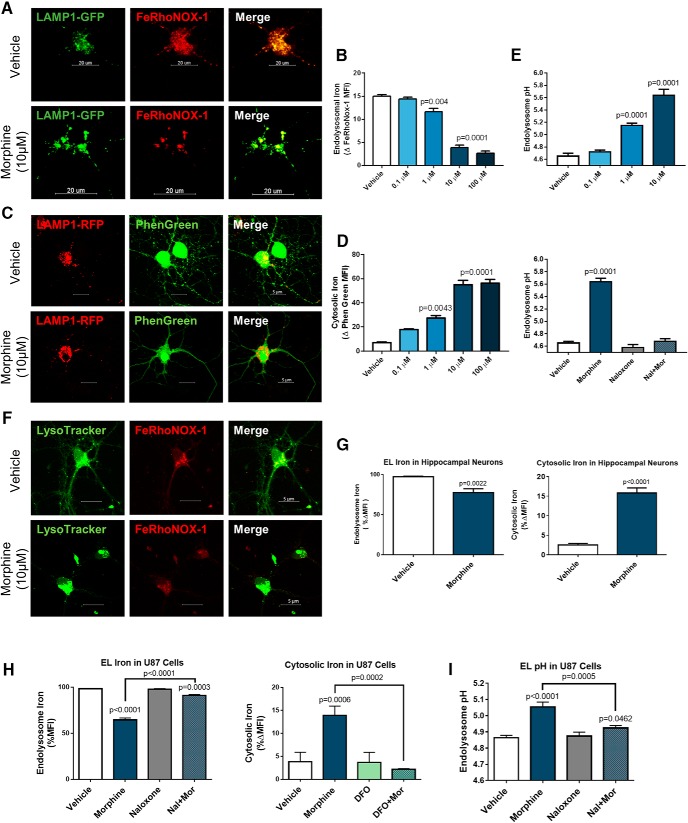
Morphine induces endolysosomal iron efflux to the cytoplasm. Visualization (***A***) and quantification (***B***) of endolysosomal iron levels in morphine-treated cortical neurons. Neurobasal cultures were transfected with LAMP1-GFP to visualize endolysosomes and loaded with the endolysosome/Golgi localized iron sensor FeRhoNox-1 (10 µM, 1 h). FeRhoNox-1 fluorescence, which is increased by iron, was measured from LAMP1-GFP-positive areas. Morphine reduced endolysosomal iron levels dose dependently, achieving statistical significance at all doses from 1 to 100 µM; *F*_(4,8)_ = 112, *p* < 0.0001. Visualization (***C***) and quantification (***D***) of cytosolic iron levels in morphine-treated cortical neurons. A different group of neurons was transfected with LAMP1-RFP and loaded with the cytoplasmically localized iron sensor phen green SK (1 µM, 30 min). Phen green fluorescence, which is quenched by iron, was measured outside of LAMP1-RFP-positive areas. Morphine increased cytosolic iron levels dose dependently, and statistical significance was achieved at all doses from 1 to 100 µM in direct agreement with endolysosomal iron studies; *F*_(4,13)_ = 47.98, *p* < 0.0001. ***E***, Morphine dose dependently de-acidifies cortical neuron endolysosomes. Neurobasal cultures were transfected with LAMP1-GFP to visualize lysosomes and loaded with pH-sensitive pHrodo dextran and pH-insensitive Alexa Fluor 647 dextran the night before drug treatments. Endolysosomal pH was calculated from the ratio of dextran emission in LAMP1-GFP-positive areas. Morphine treatment (0.1–10 µM, 30 min) increased endolysosome pH dose dependently (shown in top graph), reaching statistical significance at 1 and 10 µM doses; *F*_(3,8)_ = 180.2, *p* < 0.0001. Additionally, naloxone (50 µM) cotreatment with morphine (10 µM, 30 min) completely blocked morphine’s actions on endolysosomal pH, while naloxone alone had no effect on endolysosomal pH (shown in bottom graph); *F*_(3,8)_ = 127.2, *p* < 0.0001. All cortical neuron data were analyzed by one-way ANOVA and Dunnett *post hoc*. Iron visualization (***F***) and quantification (***G***) in morphine-treated hippocampal neurons. Hippocampal neurons were labeled with LysoTracker and FeRhoNox-1 to visualize endolysosomal iron, as shown in the micrograph. Morphine treatment (10 µM, 30 min) significantly reduced endolysosomal iron levels (*t*_(4)_ = 7.036), and increased cytoplasmic iron levels as measured by phen green SK (*t*_(4)_ = 16.86). Data analyzed by Student’s *t* test. ***H***, Iron quantification in morphine-treated U87MG cells. Endolysosomal and cytoplasmic iron levels in U87MG cells were measured with the same approach used for hippocampal neurons. Morphine (10 µM, 30 min) significantly reduced endolysosomal iron levels, which was blocked by cotreatment with naloxone (50 µM); *F*_(3,8)_ = 541.2, *p* < 0.0001. The same morphine treatment significantly increased cytoplasmic iron levels as measured by phen green SK, which was blocked by chelating endolysosomal iron with DFO (100 µM); *F*_(3,8)_ = 26.27, *p* = 0.0002. ***I***, Morphine de-acidifies endolysosomes in U87MG cells. U87MG cells were loaded with the ratiometric pH sensor Lysosensor DND-160 (1 µM, 30 min) before treatments. Morphine (10 µM, 30 min) significantly increased endolysosomal pH, which was blocked by cotreatment with naloxone; *F*_(3,8)_ = 45.05, *p* < 0.0001. U87MG data analyzed by one-way ANOVA with Tukey *post hoc*.

To determine whether morphine alters neuronal iron levels, we designed an experimental system to specifically measure free cytoplasmic iron levels, as well as iron stored in intracellular compartments. Here, we focused on endolysosomes, as they are major sites of cellular iron storage and also contain iron taken up from the extracellular space through the transferrin-mediated iron uptake pathway (Mayle et al., 2012). First, we labeled neuronal endolysosomes by transfection with either LAMP1-GFP or LAMP1-RFP (Cheng et al., 2018) depending on the iron probe used. For endolysosomal iron studies, we loaded LAMP1-GFP-transfected neurons with the endolysosome and Golgi localized iron sensor FeRhoNox-1 (Hirayama, 2018) and measured FeRhoNox-1 fluorescence exclusively in LAMP1-GFP-positive areas. After washing away excess iron sensor, we treated the cultures with morphine (0.1–100 µM, 30 min) and measured FeRhoNox-1 fluorescence immediately (Fig. 6*A*). Interestingly, morphine decreased endolysosomal iron levels dose dependently, achieving statistical significance at 1–100 µM doses (Fig. 6*B*). To determine whether morphine promoted endolysosomal iron efflux to the cytoplasm, we repeated this experiment using neurons transfected with LAMP1-RFP and loaded with the cytoplasmic iron sensor phen green SK (Hirayama, 2018), measuring phen green fluorescence outside of LAMP1-RFP-positive areas (Fig. 6*C*). As expected, morphine increased cytosolic iron levels dose dependently, also achieving statistical significance at 1–100 µM doses (Fig. 6*D*). These studies show that morphine dose dependently promotes endolysosomal iron efflux to the cytoplasm before upregulation of FHC.

Iron flux from endolysosomes may be accompanied or driven by altered pH of these compartments, which is regulated by the activity of endolysosomal iron transporters and channels (Mackenzie et al., 2007; Kiselyov et al., 2011). Therefore, we determined whether morphine altered endolysosomal pH at the same time point where we observed endolysosomal iron efflux. As before, we visualized endolysosomes by transfecting neurons with LAMP1-GFP, then loaded neurons with pH-sensitive pHrodo dextran and pH-insensitive Alexa Fluor 647 dextran. Then, we calculated the pH specifically in endolysosomes by measuring the ratio of dextran dyes within LAMP1-GFP-positive areas. Morphine de-acidified endolysosomes dose dependently, reaching statistical significance at 1 and 10 µM doses (Fig. 6*E*). This effect was completely blocked by cotreatment with the opioid antagonist naloxone (50 µM; Fig. 6*E*), showing that opioid-receptor signaling is required for endolysosome de-acidification. We also observed similar results in morphine-treated hippocampal neurons (Fig. 6*F*,*G*) and non-neuronal U87MG cells (Fig. 6*H*,*I*), suggesting that this pathway is not limited to cortical neurons.

**Figure 7.**
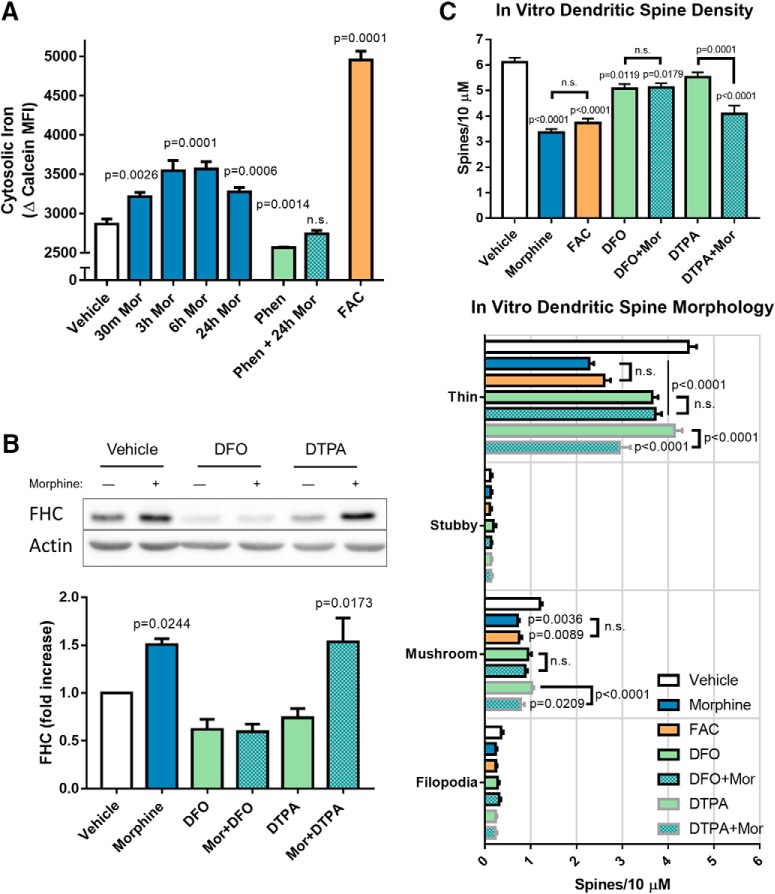
Endolysosomal iron is required for morphine-mediated FHC upregulation and reduction of mature types of dendritic spines. ***A***, Morphine increases cytoplasmic labile iron levels over 24 h in cultured neurons. Neurobasal cultures were treated with morphine (1 µM) for 30 min, 3, 6, or 24 h before loading with the cytoplasmically localized fluorescent iron sensor calcein-AM (200 nM, 30 min). Morphine treatment significantly increased cytoplasmic iron from 30 min to at least 24 h. Negative control cultures pre-treated with the iron chelator phenanthroline (10 µM, 30 min) blocked morphine's ability to increase iron levels. Positive control cultures loaded with FAC (100 µM, 24 h) significantly increased neuronal iron levels, as expected; *F*_(8,18)_ = 166.5, *p* < 0.0001. ***B***, Chelation of endolysosomal iron blocks morphine-mediated FHC upregulation. Neuronal cultures were treated with the extracellular and endolysosomal iron chelator DFO (100 µM), the cell-impermeable iron chelator DTPA (100 µM), or vehicle in combination with morphine (1 µM) and lysed 24 h later. Morphine alone significantly upregulated FHC, but DFO blocked morphine-mediated FHC upregulation. The extracellular iron chelator DTPA had no effect on morphine-mediated FHC upregulation, indicating that only intracellular iron is required for this pathway; *F*_(5,14)_ = 13.72, *p* < 0.0001. Data in ***A***, ***B*** were analyzed by one-way ANOVA and Dunnett *post hoc*. ***C***, Morphine-mediated reduction of mature dendritic spines requires endolysosomal iron. Neuronal cultures (20 DIV) were treated with morphine and various iron modulators for 24 h, followed by analysis of dendritic spine density and morphology. Morphine (1 µM) and FAC (50 µM) both significantly reduced overall dendritic spine density by the same amount. Morphine’s ability to reduce dendritic spine density was blocked by chelation of endolysosomal iron with DFO, but not affected by extracellular iron chelation with DTPA, demonstrating the importance of endolysosomal iron for this pathway; *F*_(6,56)_ = 24.21, *p* < 0.0001. Spine morphology analysis showed that morphine and FAC significantly reduced thin and mushroom spines, and this effect was similarly blocked by DFO, but not DTPA; treatment *F*_(6,224)_ = 31.61, *p* < 0.0001; spine morphology *F*_(3,224)_ = 1991, *p* < 0.0001. Spine density data were analyzed by one-way ANOVA and Tukey *post hoc*, while spine morphology data were analyzed by two-way ANOVA and Tukey *post hoc*. *N* = 3 experiments for all panels.

We followed up the previous experiments by investigating the role of iron in morphine-mediated FHC upregulation. First, we measured cytoplasmic labile iron levels in morphine-treated neuronal cultures over 24 h with the fluorescent iron sensor calcein-AM. Calcein-AM crosses the plasma membrane and interacts with cytosolic esterases that cleave the AM (acetomethoxy) residue, trapping calcein in the cytoplasm (Ma et al., 2015). We found that morphine (1 µM) significantly increased cytoplasmic iron levels as early as 30 min and out to at least 24 h (Fig. 7*A*). The slightly decreased labile iron level at 24 h post-morphine may have been due to newly translated ferritin sequestering iron away from calcein. As expected, the high concentration of FAC (100 µM, 24 h) strongly increased labile iron levels in positive control cultures, and the iron chelator phenanthroline (10 µM, 24 h) reduced labile iron below control levels and diminished morphine’s effects on labile iron levels (Fig. 7*A*). These iron loading and chelating groups also demonstrate that calcein fluorescence is sensitive to iron levels in our neuronal culture system. The prolonged increase of cytoplasmic labile iron levels after morphine treatment suggests that this mechanism drives later iron-mediated post-transcriptional upregulation of FHC in the cytoplasm.

To test this hypothesis, we next examined whether endolysosomal iron, as well as extracellular iron are necessary components of the morphine signaling pathway. We treated cultured neurons with morphine alone, and in the presence of DFO (100 µM, 24 h), which chelates endolysosomal iron (Doulias et al., 2003; Glickstein et al., 2005), or DTPA (100 µM, 24 h), which only chelates extracellular iron (Alcain et al., 1994; Mosayebnia et al., 2014). Importantly, DFO blocked morphine’s ability to upregulate FHC, while DTPA had no effect (Fig. 7*B*), suggesting that morphine’s effects on cytoplasmic labile iron levels and FHC upregulation do not depend on uptake of extracellular iron, but instead require endolysosomal iron stores.

As morphine-mediated FHC upregulation drives the reduction of mature dendritic spines, we next examined how chelation of endolysosomal and extracellular iron affected this pathway (Fig. 7*C*). As in previous dendritic spine analyses, morphine (1 µM, 24 h) significantly decreased overall dendritic spine density. Interestingly, iron loading with FAC (50 µM, 24 h), which also upregulates FHC, reduced overall dendritic spine density to a statistically indistinguishable extent as morphine. Further, morphine’s effects on spine density were blocked by endolysosomal iron chelation with DFO (100 µM, 24 h), demonstrating that endolysosomal iron is also required for morphine-mediated dendritic spine deficits. On the other hand, chelation of extracellular iron with DTPA (100 µM, 24 h) had no effect on morphine-mediated dendritic spine deficits, again suggesting that extracellular iron is not involved in this pathway. Spine morphology analyses yielded similar results, where morphine and FAC reduced mature mushroom and thin spines, and cotreatment with DFO prevented morphine’s effects, while cotreatment with DTPA did not. In line with our previous findings, these results show that morphine requires endolysosomal iron to upregulate FHC and reduce the density of mature dendritic spines, and molecules that prevent morphine-mediated FHC upregulation (CTAP, PTX, DFO) also prevent the reduction of mature dendritic spines. Taken together, these data support a novel opioid-mediated regulation of neuronal iron metabolism, which drives FHC upregulation and dendritic spine deficits in cortical neurons.

## Discussion

**Figure 8.**
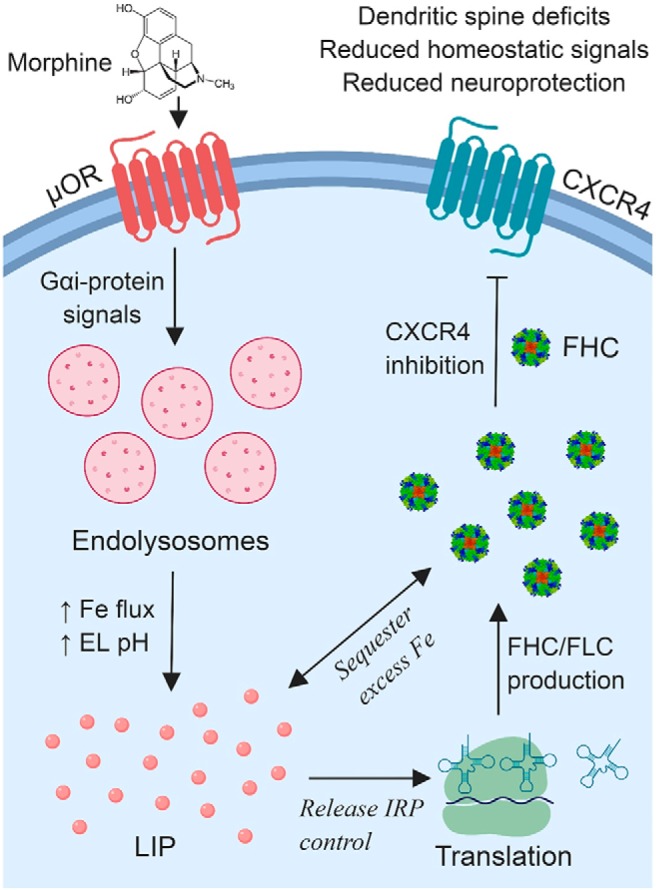
Working model of opioid regulation of FHC. Morphine-mediated activation of the µOR Gαi-protein pathway resulted in endolysosomal iron flux to the cytoplasm and a corresponding de-acidification of endolysosomes. This may be caused by µOR activation of two-pore channels (TPC), TRPML1, or DMT-1. Increased labile iron levels in the cytoplasm results in neurons producing additional FHC protein without altering FHC transcript levels. As FHC translation is controlled by IRPs that bind to FHC transcripts and prevent translation in low-iron conditions, endolysosomal iron flux may release IRPs from FHC transcripts, allowing FHC translation. FHC protein then directly interacts with the CXCR4 signaling complex and inhibits its homeostatic signaling pathways. Notably, this results in reduced dendritic spine density and reduced resilience to excitotoxicity. This pathway may be implicated in HAND with comorbid opioid use, as well as other neurologic disorders where neuronal iron levels are pathologically altered.

This study is the first to demonstrate that intracellular iron is a critical component of µ-opioid regulation of dendritic spines. We showed that µOR signaling promoted efflux of iron from endolysosomal stores to the cytoplasm, leading to the upregulation of FHC. FHC then inhibits homeostatic CXCR4 signaling and reduces the density of mature dendritic spine types in cortical neurons (Fig. 8). Thus, µ-opioid modulation of neuronal iron may be a driver that worsens cognitive impairment in HIV^+^ patients.

A recent study showed that most iron in cortical neurons is located in microsomal fractions containing the endoplasmic reticulum and vesicles (Reinert et al., 2019), suggesting that the endolysosomal system is a major iron storage site in cortical neurons, and efflux of iron from these stores can impact neuronal homeostasis. Morphine may promote iron efflux through several endolysosomal conduits, including DMT-1 (Gunshin et al., 1997), two-pore channels (Parrington et al., 2015), and TRPML1 (Dong et al., 2008; Bae et al., 2014). TRPML1 regulates endolysosomal function by releasing cations from endolysosomes, most notably calcium and iron (Dong et al., 2008; Shen et al., 2012). However, the TRPML1 agonist ML-SA1 causes endolysosomal acidification in addition to cation efflux (Bae et al., 2014), suggesting that these channels may not be involved in the morphine pathway. Two-pore channels also regulate endolysosomal function by controlling efflux of calcium and other ions (Grimm et al., 2017), but activation of these channels can result in endolysosomal de-acidification (Morgan and Galione, 2007; Pitt et al., 2014). Additionally, these channels are involved in endolysosomal iron efflux (Fernández et al., 2016). Therefore, two-pore channels are more likely to be involved in endolysosomal iron efflux after morphine treatment, but additional studies are needed to understand whether iron efflux occurs directly through these channels or by an indirect mechanism resulting from two-pore channel-mediated endolysosomal de-acidification. DMT-1 is one of the most studied iron transporters (Skjorringe et al., 2015) and another potential mediator of endolysosomal iron efflux. A recent study showed that lysosomal DMT-1 is activated in response to nNOS mediated s-nitrosylation of the small GTPase dexras1, resulting in lysosomal iron efflux (White et al., 2016). The released iron then activated a PKC and Src-dependent pathway that reduced neuronal excitability through modulation of NMDA receptors (White et al., 2016). As µOR activation can increase nNOS activity (Rodriguez-Munoz and Garzon, 2013), morphine may function through this pathway as well. Additionally, DMT-1 is an Iron-H^+^ symporter (Gunshin et al., 1997; Mackenzie et al., 2007), which may contribute to endolysosomal de-acidification as iron is exported. The acute iron-induced reduction of excitability by NMDA receptor modulation, in addition to later FHC upregulation and CXCR4 inhibition may explain the significant dendritic spine density and morphology deficits that we observed in morphine-treated cortical neurons *in vitro* and *in vivo*.

Although high labile iron levels promote oxidative stress pathways that may increase FHC transcript expression (Torti and Torti, 2002), labile iron also post-transcriptionally regulates FHC protein levels through the iron regulatory protein (IRP)/iron response element (IRE) system (Hentze and Kühn, 1996; Hentze et al., 2010). Since morphine increased labile iron levels but did not alter FHC transcript expression in our study, the IRP/IRE system may be involved in this pathway. In low-iron conditions, IRPs bind to IRE stem loop structures in the 5’ untranslated region of the FHC transcript and block its translation. In iron replete conditions where cells require additional iron storage capacity, labile iron interacts with IRPs and releases them from the FHC transcripts, promoting translation (Gray and Hentze, 1994). The IRP/IRE system regulates translation of several proteins involved in iron metabolism including FLC, and provides cells an energetically favorable way to control labile iron availability (Wilkinson and Pantopoulos, 2014). In HIV^+^ opioid users, release of IRP control coupled with inflammatory and oxidative stress response pathways that increase FHC transcript expression may further increase FHC levels, resulting in more robust CXCR4 inhibition, dendritic spine deficits, and cognitive impairment.

In addition to regulation of cytoplasmic CXCR4 signaling (Sengupta et al., 2009), FHC has distinct functions in the nucleus, including regulation of nuclear iron levels and heterochromatin stabilization (Alkhateeb and Connor, 2010). Nuclear translocation of FHC is controlled by phosphorylation of S178 (Li et al., 2006) and O-glycosylation (Surguladze et al., 2005), suggesting that an innate regulatory mechanism controls nuclear FHC protein levels. Since nuclear FHC protein expression was not affected by morphine, the mechanism controlling FHC nuclear localization may be preserved during opioid use. Additionally, the unchanged nuclear FHC levels in light of cytoplasmic upregulation may be explained by localization of iron-containing endolysosomes in morphine-treated neurons. Endolysosome de-acidification promotes a redistribution of these organelles away from perinuclear areas and toward the plasma membrane (Parton et al., 1991; Johnson et al., 2016), which could result in increased iron levels in the soma and processes and widespread FHC upregulation in the cytoplasm.

Our studies suggest that FHC inhibition of neuronal CXCR4 contributes to dendritic spine deficits (Pitcher et al., 2014). Dendritic spines exist along a continuum of maturity characterized by structural features and longevity (Berry and Nedivi, 2017). Mature spines include mushroom spines, which are the longest lasting and thought to facilitate long-term memory consolidation, and thin spines, which turnover more rapidly and facilitate short-term learning and working memory (Bourne and Harris, 2007). Spine type functions are supported by behavioral studies, suggesting that impairment in a particular facet of cognition can be predicted by dendritic spine morphology status in select brain areas, most notably hippocampus and PFC (Morrison and Baxter, 2012). Since morphine significantly decreased mushroom and thin spines in the rat medial PFC, both short and long-term memory processes may be disrupted, which could aggravate HAND symptoms. This is in agreement with our previous reports in other animal models showing cognitive impairment caused by spine loss in the same medial PFC area (Festa et al., 2015, 2018).

Individual medial PFC neurons of morphine-treated rats displayed variable FHC protein levels, suggesting that morphine’s actions may be driven by specific neuronal subpopulations. µOR-expressing neurons are obvious candidates, since FHC upregulation, as well as dendritic spine deficits are completely blocked by the µOR antagonist CTAP (Sengupta et al., 2009). Previous evidence suggests that µORs may be expressed in excitatory cortical neurons *in vitro* (Liao et al., 2005; Patel et al., 2006), indicating that morphine may directly upregulate FHC and reduce dendritic spine density in the same neuron. However, other reports suggest that µORs are strongly expressed in rat cortical GABAergic interneurons (Taki et al., 2000; Férézou et al., 2007). Therefore, morphine modulation of interneuron activity could lead to FHC upregulation and dendritic spine deficits in nearby excitatory neurons. Additionally, morphine may directly upregulate FHC and inhibit CXCR4 in µOR-expressing cortical interneurons. As CXCR4 regulates GABAergic transmission in several brain areas (Guyon, 2014; Wu et al., 2017), disruption of this pathway could also lead to altered inhibition, FHC upregulation, and dendritic spine deficits in excitatory neurons. Select cortical interneurons and excitatory neurons also express CXCR4 (Banisadr et al., 2002), suggesting that all of these scenarios could potentially occur. Morphine’s actions on cortical inhibition may also differ depending on the type of interneuron affected. FHC upregulation or dampening of µOR expressing 5HT3A and vasoactive intestinal peptide disinhibitory interneurons may disrupt their tuning of inhibition in the cortex (Karnani et al., 2016). Furthermore, FHC upregulation or dampening of µOR expressing parvalbumin, somatostatin, or cholecystokinin interneurons may disrupt their ability to tune the excitation of nearby excitatory or inhibitory neurons (Artinian and Lacaille, 2018; Ferguson and Gao, 2018; Whissell et al., 2019). Therefore, altered GABAergic transmission, which is an important component of HAND pathology (Buzhdygan et al., 2016; Marks et al., 2016; Xu and Fitting, 2016), may be further disrupted by opioids.

Neuronal iron plays a crucial role in cognition across neurocognitive disease states (Rouault, 2013; Dusek et al., 2016; Zhou and Tan, 2017), and iron chelation therapy for neurocognitive disorders has been reported with positive results (Crapper McLachlan et al., 1991; Devos et al., 2014; Wang et al., 2015; Dusek et al., 2016; Crielaard et al., 2017). However, more work is needed to determine its value as a therapeutic approach for HAND with comorbid opioid use. In humans, neuronal FHC upregulation is caused by chronic low-level immune activation and aggravated by opioids, which may partially explain faster disease progression in opioid drug abusers. Targeted iron chelation may be effective at reversing morphine-mediated FHC upregulation, but it remains to be established whether it would also affect IL-1β and TNFα-mediated FHC upregulation (Festa et al., 2015).

Overall, these studies uncovered a novel interaction of morphine with the endogenous iron regulatory system and point to a key role of endolysosomes in the induction of neuronal FHC expression by morphine, which has important consequences on neuronal connectivity. Furthermore, our findings in non-neuronal cells indicate that this mechanism may not be limited to neurons, suggesting a broader impact of the interaction between opioids and cellular iron.

## Synthesis

Reviewing Editor: Karen Szumlinski, University of California at Santa Barbara Decisions are customarily a result of the Reviewing Editor and the peer reviewers coming together and discussing their recommendations until a consensus is reached. When revisions are invited, a fact-based synthesis statement explaining their decision and outlining what is needed to prepare a revision will be listed below. Note: If this manuscript was transferred from JNeurosci and a decision was made to accept the manuscript without peer review, a brief statement to this effect will instead be what is listed below.

In reviewing the authors' rebuttal and the revised version of this manuscript, forwarded from JNS, I am pleased to extend the decision of “Accept” as I found the data novel and compelling. In my opinion, the authors did an excellent job of addressing the prior comments and I agree with the authors that requesting further experiments at this time would unnecessarily delay the publication of these new findings linking morphine-induced changes in iron and ferritin in relation to dendritic spine morphology. I trust that the authors will continue to develop their story and that future work will address some of the lingering issues raised by Reviewer 1 of the JNS version of the article.

## References

[B1] Alcain FJ, Löw H, Crane FL (1994) Iron reverses impermeable chelator inhibition of DNA synthesis in CCl 39 cells. Proc Natl Acad Sci USA 91:7903–7906. 10.1073/pnas.91.17.7903 8058732 PMC44512

[B2] Alkhateeb AA, Connor JR (2010) Nuclear ferritin: a new role for ferritin in cell biology. Biochim Biophys Acta 1800:793–797. 10.1016/j.bbagen.2010.03.017 20347012

[B3] Artinian J, Lacaille JC (2018) Disinhibition in learning and memory circuits: new vistas for somatostatin interneurons and long-term synaptic plasticity. Brain Res Bull 141:20–26. 10.1016/j.brainresbull.2017.11.012 29174732

[B4] Bae M, Patel N, Xu H, Lee M, Tominaga-Yamanaka K, Nath A, Geiger J, Gorospe M, Mattson MP, Haughey NJ (2014) Activation of TRPML1 clears intraneuronal Aβ in preclinical models of HIV infection. J Neurosci 34:11485–11503. 10.1523/JNEUROSCI.0210-14.2014 25143627 PMC4138351

[B5] Bandaru VV, Patel N, Ewaleifoh O, Haughey NJ (2011) A failure to normalize biochemical and metabolic insults during morphine withdrawal disrupts synaptic repair in mice transgenic for HIV-gp120. J Neuroimmune Pharmacol 6:640–649. 10.1007/s11481-011-9289-0 21748284 PMC3422763

[B6] Banisadr G, Fontanges P, Haour F, Kitabgi P, Rostène W, Mélik Parsadaniantz S (2002) Neuroanatomical distribution of CXCR4 in adult rat brain and its localization in cholinergic and dopaminergic neurons. Eur J Neurosci 16:1661–1671. 12431218 10.1046/j.1460-9568.2002.02237.x

[B7] Berry KP, Nedivi E (2017) Spine dynamics: are they all the same? Neuron 96:43–55. 10.1016/j.neuron.2017.08.008 28957675 PMC5661952

[B8] Bloss EB, Janssen WG, Ohm DT, Yuk FJ, Wadsworth S, Saardi KM, McEwen BS, Morrison JH (2011) Evidence for reduced experience-dependent dendritic spine plasticity in the aging prefrontal cortex. J Neurosci 31:7831–7839. 10.1523/JNEUROSCI.0839-11.2011 21613496 PMC3398699

[B9] Bohn LM, Lefkowitz RJ, Gainetdinov RR, Peppel K, Caron MG, Lin FT (1999) Enhanced morphine analgesia in mice lacking beta-arrestin 2. Science 286:2495–2498. 10.1126/science.286.5449.2495 10617462

[B10] Bourne J, Harris KM (2007) Do thin spines learn to be mushroom spines that remember? Curr Opin Neurobiol 17:381–386. 10.1016/j.conb.2007.04.009 17498943

[B11] Breuer W, Epsztejn S, Millgram P, Cabantchik IZ (1995) Transport of iron and other transition metals into cells as revealed by a fluorescent probe. Am J Physiol 268:C1354–C1361. 10.1152/ajpcell.1995.268.6.C1354 7611353

[B12] Brewer GJ, Torricelli JR, Evege EK, Price PJ (1993) Optimized survival of hippocampal neurons in B27-supplemented Neurobasal, a new serum-free medium combination. J Neurosci Res 35:567–576. 10.1002/jnr.490350513 8377226

[B13] Burbassi S, Sengupta R, Meucci O (2010) Alterations of CXCR4 function in µ-opioid receptor-deficient glia. Eur J Neurosci 32:1278–1288. 10.1111/j.1460-9568.2010.07402.x 20880358 PMC2956872

[B14] Buzhdygan T, Lisinicchia J, Patel V, Johnson K, Neugebauer V, Paessler S, Jennings K, Gelman B (2016) Neuropsychological, neurovirological and neuroimmune aspects of abnormal GABAergic transmission in HIV infection. J Neuroimmune Pharmacol 11:279–293. 10.1007/s11481-016-9652-2 26829944 PMC4848342

[B15] Byrd DA, Fellows RP, Morgello S, Franklin D, Heaton RK, Deutsch R, Atkinson JH, Clifford DB, Collier AC, Marra CM, Gelman B, McCutchan JA, Duarte NA, Simpson DM, McArthur J, Grant I; CHARTER Group (2011) Neurocognitive impact of substance use in HIV infection. J Acquir Immune Defic Syndr 58:154–162. 10.1097/QAI.0b013e318229ba41 21725250 PMC3183737

[B16] Cheepsunthorn P, Palmer C, Connor JR (1998) Cellular distribution of ferritin subunits in postnatal rat brain. J Comp Neurol 400:73–86. 9762867

[B17] Chen X, Geller EB, Rogers TJ, Adler MW (2007) Rapid heterologous desensitization of antinociceptive activity between mu or delta opioid receptors and chemokine receptors in rats. Drug Alcohol Depend 88:36–41. 10.1016/j.drugalcdep.2006.09.010 17049756 PMC1880888

[B18] Cheng XT, Xie YX, Zhou B, Huang N, Farfel-Becker T, Sheng ZH (2018) Characterization of LAMP1-labeled nondegradative lysosomal and endocytic compartments in neurons. J Cell Biol 217:3127–3139. 10.1083/jcb.201711083 29695488 PMC6123004

[B19] Chiazza F, Tammen H, Pintana H, Lietzau G, Collino M, Nyström T, Klein T, Darsalia V, Patrone C (2018) The effect of DPP-4 inhibition to improve functional outcome after stroke is mediated by the SDF-1α/CXCR4 pathway. Cardiovasc Diabetol 17:60. 10.1186/s12933-018-0702-3 29776406 PMC5960142

[B20] Cozzi A, Corsi B, Levi S, Santambrogio P, Albertini A, Arosio P (2000) Overexpression of wild type and mutated human ferritin H-chain in HeLa cells: in vivo role of ferritin ferroxidase activity. J Biol Chem 275:25122–25129. 10.1074/jbc.M003797200 10833524

[B21] Crapper McLachlan DR, Dalton AJ, Kruck TP, Bell MY, Smith WL, Kalow W, Andrews DF (1991) Intramuscular desferrioxamine in patients with Alzheimer's disease. Lancet 337:1304–1308. 10.1016/0140-6736(91)92978-B 1674295

[B22] Crielaard BJ, Lammers T, Rivella S (2017) Targeting iron metabolism in drug discovery and delivery. Nat Rev Drug Discov 16:400–423. 10.1038/nrd.2016.248 28154410 PMC5455971

[B23] Devos D, Moreau C, Devedjian JC, Kluza J, Petrault M, Laloux C, Jonneaux A, Ryckewaert G, Garçon G, Rouaix N, Duhamel A, Jissendi P, Dujardin K, Auger F, Ravasi L, Hopes L, Grolez G, Firdaus W, Sablonnière B, Strubi-Vuillaume I, et al. (2014) Targeting chelatable iron as a therapeutic modality in Parkinson's disease. Antioxid Redox Signal 21:195–210. 10.1089/ars.2013.5593 24251381 PMC4060813

[B24] Di Prisco S, Olivero G, Merega E, Bonfiglio T, Marchi M, Pittaluga A (2016) CXCR4 and NMDA receptors are functionally coupled in rat hippocampal noradrenergic and glutamatergic nerve endings. J Neuroimmune Pharmacol 11:645–656. 10.1007/s11481-016-9677-6 27147258

[B25] Dong XP, Cheng X, Mills E, Delling M, Wang F, Kurz T, Xu H (2008) The type IV mucolipidosis-associated protein TRPML1 is an endolysosomal iron release channel. Nature 455:992–996. 10.1038/nature07311 18794901 PMC4301259

[B26] Doulias PT, Christoforidis S, Brunk UT, Galaris D (2003) Endosomal and lysosomal effects of desferrioxamine: protection of HeLa cells from hydrogen peroxide-induced DNA damage and induction of cell-cycle arrest. Free Radic Biol Med 35:719–728. 14583336 10.1016/s0891-5849(03)00396-4

[B27] Dumitriu D, Hao J, Hara Y, Kaufmann J, Janssen WG, Lou W, Rapp PR, Morrison JH (2010) Selective changes in thin spine density and morphology in monkey prefrontal cortex correlate with aging-related cognitive impairment. J Neurosci 30:7507–7515. 10.1523/JNEUROSCI.6410-09.2010 20519525 PMC2892969

[B28] Dusek P, Schneider SA, Aaseth J (2016) Iron chelation in the treatment of neurodegenerative diseases. J Trace Elem Med Biol 38:81–92. 27033472 10.1016/j.jtemb.2016.03.010

[B29] El-Hage N, Rodriguez M, Dever SM, Masvekar RR, Gewirtz DA, Shacka JJ (2015) HIV-1 and morphine regulation of autophagy in microglia: limited interactions in the context of HIV-1 infection and opioid abuse. J Virol 89:1024–1035. 10.1128/JVI.02022-14 25355898 PMC4300622

[B30] Epsztejn S, Kakhlon O, Glickstein H, Breuer W, Cabantchik I (1997) Fluorescence analysis of the labile iron pool of mammalian cells. Anal Biochem 248:31–40. 10.1006/abio.1997.2126 9177722

[B31] Espósito BP, Epsztejn S, Breuer W, Cabantchik ZI (2002) A review of fluorescence methods for assessing labile iron in cells and biological fluids. Anal Biochem 304:1–18. 10.1006/abio.2002.5611 11969183

[B32] Férézou I, Hill EL, Cauli B, Gibelin N, Kaneko T, Rossier J, Lambolez B (2007) Extensive overlap of mu-opioid and nicotinic sensitivity in cortical interneurons. Cereb Cortex 17:1948–1957. 10.1093/cercor/bhl104 17068095

[B33] Ferguson BR, Gao WJ (2018) PV interneurons: critical regulators of E/I balance for prefrontal cortex-dependent behavior and psychiatric disorders. Front Neural Circuits 12:37. 10.3389/fncir.2018.00037 29867371 PMC5964203

[B34] Fernández B, Fdez E, Gómez-Suaga P, Gil F, Molina-Villalba I, Ferrer I, Patel S, Churchill GC, Hilfiker S (2016) Iron overload causes endolysosomal deficits modulated by NAADP-regulated 2-pore channels and RAB7A. Autophagy 12:1487–1506. 10.1080/15548627.2016.1190072 27383256 PMC5082776

[B35] Festa L, Gutoskey CJ, Graziano A, Waterhouse BD, Meucci O (2015) Induction of interleukin-1β by human immunodeficiency virus-1 viral proteins leads to increased levels of neuronal ferritin heavy chain, synaptic injury, and deficits in flexible attention. J Neurosci 35:10550–10561. 10.1523/JNEUROSCI.4403-14.2015 26203149 PMC4510293

[B36] Festa L, Platt B, Tian Y, Floresco S, Meucci O (2018) Abstracts from the joint meeting of the International Society for NeuroVirology (ISNV) and the Society on NeuroImmune Pharmacology (SNIP) April 10-14, 2018, Chicago, Illinois, USA. J Neuroimmune Pharmacol 13:1–102. 10.1007/s11481-018-9786-5 29582223

[B37] Fitting S, Knapp PE, Zou S, Marks WD, Bowers MS, Akbarali HI, Hauser KF (2014) Interactive HIV-1 Tat and morphine-induced synaptodendritic injury is triggered through focal disruptions in Na(+) influx, mitochondrial instability, and Ca(2)(+) overload. J Neurosci 34:12850–12864. 10.1523/JNEUROSCI.5351-13.2014 25232120 PMC4166164

[B38] Glickstein H, El RB, Shvartsman M, Cabantchik ZI (2005) Intracellular labile iron pools as direct targets of iron chelators: a fluorescence study of chelator action in living cells. Blood 106:3242–3250. 10.1182/blood-2005-02-0460 16020512

[B39] Gray NK, Hentze MW (1994) Iron regulatory protein prevents binding of the 43S translation pre-initiation complex to ferritin and eALAS mRNAs. EMBO J 13:3882–3891. 8070415 10.1002/j.1460-2075.1994.tb06699.x PMC395301

[B40] Grimm C, Chen CC, Wahl-Schott C, Biel M (2017) Two-pore channels: catalyzers of endolysosomal transport and function. Front Pharmacol 8:45. 10.3389/fphar.2017.00045 28223936 PMC5293812

[B41] Gunshin H, Mackenzie B, Berger UV, Gunshin Y, Romero MF, Boron WF, Nussberger S, Gollan JL, Hediger MA (1997) Cloning and characterization of a mammalian proton-coupled metal-ion transporter. Nature 388:482–488. 10.1038/41343 9242408

[B42] Guo M, Bryant J, Sultana S, Jones O, Royal W 3rd (2012) Effects of vitamin A deficiency and opioids on parvalbumin + interneurons in the hippocampus of the HIV-1 transgenic rat. Curr HIV Res 10:463–468. 22591370 10.2174/157016212802138715 PMC4057432

[B43] Guyon A (2014) CXCL12 chemokine and GABA neurotransmitter systems crosstalk and their putative roles. Front Cell Neurosci 5:115. 10.3389/fncel.2014.00115 24808825 PMC4009426

[B44] Guyon A, Nahon JL (2007) Multiple actions of the chemokine stromal cell-derived factor-1alpha on neuronal activity. J Mol Endocrinol 38:365–376. 10.1677/JME-06-0013 17339399

[B45] Hentze MW, Kühn LC (1996) Molecular control of vertebrate iron metabolism: mRNA-based regulatory circuits operated by iron, nitric oxide, and oxidative stress. Proc Natl Acad Sci USA 93:8175–8182. 10.1073/pnas.93.16.8175 8710843 PMC38642

[B46] Hentze MW, Rouault TA, Caughman SW, Dancis A, Harford JB, Klausner RD (1987) A cis-acting element is necessary and sufficient for translational regulation of human ferritin expression in response to iron. Proc Natl Acad Sci USA 84:6730–6734. 10.1073/pnas.84.19.6730 3477805 PMC299157

[B47] Hentze MW, Muckenthaler MU, Galy B, Camaschella C (2010) Two to tango: regulation of mammalian iron metabolism. Cell 142:24–38. 10.1016/j.cell.2010.06.028 20603012

[B48] Hirayama T (2018) Development of chemical tools for imaging of Fe(II) ions in living cells: a review. Acta Histochem Cytochem 51:137–143. 10.1267/ahc.18015 30510327 PMC6261839

[B49] Hirayama T, Okuda K, Nagasawa H (2013) A highly selective turn-on fluorescent probe for iron(ii) to visualize labile iron in living cells. Chem Sci 4:1250 10.1039/c2sc21649c

[B50] Hui L, Chen X, Haughey NJ, Geiger JD (2012) Role of endolysosomes in HIV-1 Tat-induced neurotoxicity. ASN Neuro 4:243–252. 10.1042/AN20120017 22591512 PMC3379000

[B51] Johnson DE, Ostrowski P, Jaumouillé V, Grinstein S (2016) The position of lysosomes within the cell determines their luminal pH. J Cell Biol 212:677–692. 10.1083/jcb.201507112 26975849 PMC4792074

[B52] Kallianpur AR, Gittleman H, Letendre S, Ellis R, Barnholtz-Sloan JS, Bush WS, Heaton R, Samuels DC, Franklin DR Jr, Rosario-Cookson D, Clifford DB, Collier AC, Gelman B, Marra CM, McArthur JC, McCutchan JA, Morgello S, Grant I, Simpson D, Connor JR, et al. (2019) Cerebrospinal fluid ceruloplasmin, haptoglobin, and vascular endothelial growth factor are associated with neurocognitive impairment in adults with HIV infection. Mol Neurobiol 56:3808–3818. 10.1007/s12035-018-1329-9 30209774 PMC6952215

[B53] Karnani MM, Jackson J, Ayzenshtat I, Hamzehei Sichani A, Manoocheri K, Kim S, Yuste R (2016) Opening holes in the blanket of inhibition: localized lateral disinhibition by VIP interneurons. J Neurosci 36:3471–3480. 10.1523/JNEUROSCI.3646-15.2016 27013676 PMC4804006

[B54] Khan MZ, Brandimarti R, Musser BJ, Resue DM, Fatatis A, Meucci O (2003) The chemokine receptor CXCR4 regulates cell-cycle proteins in neurons. J Neurovirol 9:300–314. 10.1080/13550280390201010 12775414 PMC2669737

[B55] Khan MZ, Brandimarti R, Shimizu S, Nicolai J, Crowe E, Meucci O (2008) The chemokine CXCL12 promotes survival of postmitotic neurons by regulating Rb protein. Cell Death Differ 15:1663–1672. 10.1038/cdd.2008.95 18583990 PMC2676689

[B56] Kiselyov K, Colletti GA, Terwilliger A, Ketchum K, Lyons CW, Quinn J, Muallem S (2011) TRPML: transporters of metals in lysosomes essential for cell survival? Cell Calcium 50:288–294. 10.1016/j.ceca.2011.04.009 21621258 PMC3164942

[B57] Knovich MA, Storey JA, Coffman LG, Torti SV, Torti FM (2009) Ferritin for the clinician. Blood Rev 23:95–104. 10.1016/j.blre.2008.08.001 18835072 PMC2717717

[B58] Kokovay E, Goderie S, Wang Y, Lotz S, Lin G, Sun Y, Roysam B, Shen Q, Temple S (2010) Adult SVZ lineage cells home to and leave the vascular niche via differential responses to SDF1/CXCR4 signaling. Cell Stem Cell 7:163–173. 10.1016/j.stem.2010.05.019 20682445 PMC2916873

[B59] Leibold EA, Munro HN (1988) Cytoplasmic protein binds in vitro to a highly conserved sequence in the 5' untranslated region of ferritin heavy- and light-subunit mRNAs. Proc Natl Acad Sci USA 85:2171–2175. 10.1073/pnas.85.7.2171 3127826 PMC279951

[B60] Li R, Luo C, Mines M, Zhang J, Fan GH (2006) Chemokine CXCL12 induces binding of ferritin heavy chain to the chemokine receptor CXCR4, alters CXCR4 signaling, and induces phosphorylation and nuclear translocation of ferritin heavy chain. J Biol Chem 281:37616–37627. 10.1074/jbc.M607266200 17056593

[B61] Liao D, Lin H, Law PY, Loh HH (2005) Mu-opioid receptors modulate the stability of dendritic spines. Proc Natl Acad Sci USA 102:1725–1730. 10.1073/pnas.0406797102 15659552 PMC545084

[B62] Ma Y, Abbate V, Hider RC (2015) Iron-sensitive fluorescent probes: monitoring intracellular iron pools. Metallomics 7:212–222. 10.1039/c4mt00214h 25315476

[B63] Mackenzie B, Takanaga H, Hubert N, Rolfs A, Hediger MA (2007) Functional properties of multiple isoforms of human divalent metal-ion transporter 1 (DMT1). Biochem J 403:59–69. 10.1042/BJ20061290 17109629 PMC1828886

[B64] Manglik A, Lin H, Aryal DK, McCorvy JD, Dengler D, Corder G, Levit A, Kling RC, Bernat V, Hubner H, Huang XP, Sassano MF, Giguere PM, Lober S, Da D, Scherrer G, Kobilka BK, Gmeiner P, Roth BL, Shoichet BK (2016) Structure-based discovery of opioid analgesics with reduced side effects. Nature 537:185–190. 27533032 10.1038/nature19112 PMC5161585

[B65] Marks WD, Paris JJ, Schier CJ, Denton MD, Fitting S, McQuiston AR, Knapp PE, Hauser KF (2016) HIV-1 Tat causes cognitive deficits and selective loss of parvalbumin, somatostatin, and neuronal nitric oxide synthase expressing hippocampal CA1 interneuron subpopulations. J Neurovirol 22:747–762. 10.1007/s13365-016-0447-2 27178324 PMC5107352

[B66] Mayle KM, Le AM, Kamei DT (2012) The intracellular trafficking pathway of transferrin. Biochim Biophys Acta 1820:264–281. 10.1016/j.bbagen.2011.09.009 21968002 PMC3288267

[B67] McArthur JC, Steiner J, Sacktor N, Nath A (2010) Human immunodeficiency virus-associated neurocognitive disorders: mind the gap. Ann Neurol 67:699–714. 10.1002/ana.22053 20517932

[B68] McGuire JL, Barrett JS, Vezina HE, Spitsin S, Douglas SD (2014) Adjuvant therapies for HIV-associated neurocognitive disorders. Ann Clin Transl Neurol 1:938–952. 10.1002/acn3.131 25540809 PMC4265066

[B69] Meucci O, Fatatis A, Simen AA, Bushell TJ, Gray PW, Miller RJ (1998) Chemokines regulate hippocampal neuronal signaling and gp120 neurotoxicity. Proc Natl Acad Sci USA 95:14500–14505. 10.1073/pnas.95.24.14500 9826729 PMC24402

[B70] Morgan AJ, Galione A (2007) NAADP induces pH changes in the lumen of acidic Ca2+ stores. Biochem J 402:301–310. 10.1042/BJ20060759 17117921 PMC1798430

[B71] Morrison JH, Baxter MG (2012) The ageing cortical synapse: hallmarks and implications for cognitive decline. Nat Rev Neurosci 13:240–250. 10.1038/nrn3200 22395804 PMC3592200

[B72] Mosayebnia M, Shafiee-Ardestani M, Pasalar P, Mashayekhi M, Amanlou M (2014) Diethylentriaminepenta acetic acid glucose conjugates as a cell permeable iron chelator. J Pharmacol Pharmacother 5:27–32. 10.4103/0976-500X.124416 24554907 PMC3917162

[B73] Mukaide T, Hattori Y, Misawa N, Funahashi S, Jiang L, Hirayama T, Nagasawa H, Toyokuni S (2014) Histological detection of catalytic ferrous iron with the selective turn-on fluorescent probe RhoNox-1 in a Fenton reaction-based rat renal carcinogenesis model. Free Radic Res 48:990–995. 10.3109/10715762.2014.898844 24580501

[B74] Nash B, Meucci O (2014) Functions of the chemokine receptor CXCR4 in the central nervous system and its regulation by µ-opioid receptors. Int Rev Neurobiol 118:105–128. 10.1016/B978-0-12-801284-0.00005-1 25175863 PMC4369781

[B75] Nicolai J, Burbassi S, Rubin J, Meucci O (2010) CXCL12 inhibits expression of the NMDA receptor's NR2B subunit through a histone deacetylase-dependent pathway contributing to neuronal survival. Cell Death Dis 1:e33. 10.1038/cddis.2010.10 21364640 PMC3032300

[B76] Parrington J, Lear P, Hachem A (2015) Calcium signals regulated by NAADP and two-pore channels–their role in development, differentiation and cancer. Int J Dev Biol 59:341–355. 10.1387/ijdb.150211jp 26679949

[B77] Parton RG, Dotti CG, Bacallao R, Kurtz I, Simons K, Prydz K (1991) pH-induced microtubule-dependent redistribution of late endosomes in neuronal and epithelial cells. J Cell Biol 113:261–274. 10.1083/jcb.113.2.261 2010463 PMC2288934

[B78] Patel JP, Sengupta R, Bardi G, Khan MZ, Mullen-Przeworski A, Meucci O (2006) Modulation of neuronal CXCR4 by the micro-opioid agonist DAMGO. J Neurovirol 12:492–500. 10.1080/13550280601064798 17162664 PMC2676683

[B79] Patton SM, Wang Q, Hulgan T, Connor JR, Jia P, Zhao Z, Letendre SL, Ellis RJ, Bush WS, Samuels DC, Franklin DR, Kaur H, Iudicello J, Grant I, Kallianpur AR (2017) Cerebrospinal fluid (CSF) biomarkers of iron status are associated with CSF viral load, antiretroviral therapy, and demographic factors in HIV-infected adults. Fluids Barriers CNS 14:11. 10.1186/s12987-017-0058-1 28427421 PMC5399327

[B80] Pello OM, Martínez-Muñoz L, Parrillas V, Serrano A, Rodríguez-Frade JM, Toro MJ, Lucas P, Monterrubio M, Martínez AC, Mellado M (2008) Ligand stabilization of CXCR4/delta-opioid receptor heterodimers reveals a mechanism for immune response regulation. Eur J Immunol 38:537–549. 10.1002/eji.200737630 18200497

[B81] Petrat F, Rauen U, de Groot H (1999) Determination of the chelatable iron pool of isolated rat hepatocytes by digital fluorescence microscopy using the fluorescent probe, phen green SK. Hepatology 29:1171–1179. 10.1002/hep.510290435 10094962

[B82] Petrat F, de Groot H, Rauen U (2001) Subcellular distribution of chelatable iron: a laser scanning microscopic study in isolated hepatocytes and liver endothelial cells. Biochem J 356:61–69. 10.1042/0264-6021:3560061 11336636 PMC1221812

[B83] Pitcher J, Wurth R, Shimizu S, Meucci O (2013) Multispectral imaging and automated laser capture microdissection of human cortical neurons: a quantitative study of CXCR4 expression. Methods Mol Biol 1013:31–48. 10.1007/978-1-62703-426-5_3 23625491 PMC4011070

[B84] Pitcher J, Abt A, Myers J, Han R, Snyder M, Graziano A, Festa L, Kutzler M, Garcia F, Gao WJ, Fischer-Smith T, Rappaport J, Meucci O (2014) Neuronal ferritin heavy chain and drug abuse affect HIV-associated cognitive dysfunction. J Clin Invest 124:656–669. 10.1172/JCI70090 24401274 PMC3904611

[B85] Pitt SJ, Lam AK, Rietdorf K, Galione A, Sitsapesan R (2014) Reconstituted human TPC1 is a proton-permeable ion channel and is activated by NAADP or Ca2+. Sci Signal 7:ra46. 10.1126/scisignal.2004854 24847115 PMC6669042

[B86] Rauen U, Kerkweg U, Weisheit D, Petrat F, Sustmann R, de Groot H (2003) Cold-induced apoptosis of hepatocytes: mitochondrial permeability transition triggered by nonmitochondrial chelatable iron. Free Radic Biol Med 35:1664–1678. 14680689 10.1016/j.freeradbiomed.2003.09.018

[B87] Reinert A, Morawski M, Seeger J, Arendt T, Reinert T (2019) Iron concentrations in neurons and glial cells with estimates on ferritin concentrations. BMC Neurosci 20:25. 10.1186/s12868-019-0507-7 31142282 PMC6542065

[B88] Reynolds IJ (2004) Fluorescence detection of redox-sensitive metals in neuronal culture: focus on iron and zinc. Ann NY Acad Sci 1012:27–36. 10.1196/annals.1306.003 15105253

[B89] Rodriguez-Munoz M, Garzon J (2013) Nitric oxide and zinc-mediated protein assemblies involved in mu opioid receptor signaling. Mol Neurobiol 48:769–782. 23666425 10.1007/s12035-013-8465-z

[B90] Rodriguez A, Ehlenberger DB, Dickstein DL, Hof PR, Wearne SL (2008) Automated three-dimensional detection and shape classification of dendritic spines from fluorescence microscopy images. PLoS One 3:e1997. 10.1371/journal.pone.0001997 18431482 PMC2292261

[B91] Rouault TA (2013) Iron metabolism in the CNS: implications for neurodegenerative diseases. Nat Rev Neurosci 14:551–564. 10.1038/nrn3453 23820773

[B92] Sanchez-Blazquez P, Gomez-Serranillos P, Garzon J (2001) Agonists determine the pattern of G-protein activation in mu-opioid receptor-mediated supraspinal analgesia. Brain Res Bull 54:229–235. 11275413 10.1016/s0361-9230(00)00448-2

[B93] Saylor D, Dickens AM, Sacktor N, Haughey N, Slusher B, Pletnikov M, Mankowski JL, Brown A, Volsky DJ, McArthur JC (2016) HIV-associated neurocognitive disorder - pathogenesis and prospects for treatment. Nat Rev Neurol 12:309. 10.1038/nrneurol.2016.53 27080521 PMC5842923

[B94] Schmid CL, Kennedy NM, Ross NC, Lovell KM, Yue Z, Morgenweck J, Cameron MD, Bannister TD, Bohn LM (2017) Bias factor and therapeutic window correlate to predict safer opioid analgesics. Cell 171:1165–1175.e13. 10.1016/j.cell.2017.10.035 29149605 PMC5731250

[B95] Seabold GK, Daunais JB, Rau A, Grant KA, Alvarez VA (2010) DiOLISTIC labeling of neurons from rodent and non-human primate brain slices. J Vis Exp (41). 10.3791/2081 PMC3156079 20644510

[B96] Sengupta R, Burbassi S, Shimizu S, Cappello S, Vallee RB, Rubin JB, Meucci O (2009) Morphine increases brain levels of ferritin heavy chain leading to inhibition of CXCR4-mediated survival signaling in neurons. J Neurosci 29:2534–2544. 10.1523/JNEUROSCI.5865-08.2009 19244528 PMC2664553

[B97] Shen D, Wang X, Li X, Zhang X, Yao Z, Dibble S, Dong XP, Yu T, Lieberman AP, Showalter HD, Xu H (2012) Lipid storage disorders block lysosomal trafficking by inhibiting a TRP channel and lysosomal calcium release. Nat Commun 3:731. 10.1038/ncomms1735 22415822 PMC3347486

[B98] Shepherd AJ, Loo L, Mohapatra DP (2013) Chemokine co-receptor CCR5/CXCR4-dependent modulation of Kv2.1 channel confers acute neuroprotection to HIV-1 glycoprotein gp120 exposure. PLoS One 8:e76698. 10.1371/journal.pone.0076698 24086760 PMC3782454

[B99] Shimizu S, Abt A, Meucci O (2011a) Bilaminar co-culture of primary rat cortical neurons and glia. J Vis Exp. Advance online publication. Retrieved November 12, 2011. doi: 10.3791/3257. 10.3791/3257 PMC3308589 22105098

[B100] Shimizu S, Brown M, Sengupta R, Penfold ME, Meucci O (2011b) CXCR7 protein expression in human adult brain and differentiated neurons. PLoS One 6:e20680 10.1371/journal.pone.0020680 21655198 PMC3105114

[B101] Skjorringe T, Burkhart A, Johnsen KB, Moos T (2015) Divalent metal transporter 1 (DMT1) in the brain: implications for a role in iron transport at the blood-brain barrier, and neuronal and glial pathology. Front Mol Neurosci 8:19. 26106291 10.3389/fnmol.2015.00019 PMC4458610

[B102] Soergel DG, Subach RA, Burnham N, Lark MW, James IE, Sadler BM, Skobieranda F, Violin JD, Webster LR (2014) Biased agonism of the µ-opioid receptor by TRV130 increases analgesia and reduces on-target adverse effects versus morphine: a randomized, double-blind, placebo-controlled, crossover study in healthy volunteers. Pain 155:1829–1835. 10.1016/j.pain.2014.06.011 24954166

[B103] Storr HL, Kind B, Parfitt DA, Chapple JP, Lorenz M, Koehler K, Huebner A, Clark AJ (2009) Deficiency of ferritin heavy-chain nuclear import in triple a syndrome implies nuclear oxidative damage as the primary disease mechanism. Mol Endocrinol 23:2086–2094. 10.1210/me.2009-0056 19855093 PMC5419132

[B104] Surguladze N, Thompson KM, Beard JL, Connor JR, Fried MG (2004) Interactions and reactions of ferritin with DNA. J Biol Chem 279:14694–14702. 10.1074/jbc.M313348200 14734543

[B105] Surguladze N, Patton S, Cozzi A, Fried MG, Connor JR (2005) Characterization of nuclear ferritin and mechanism of translocation. Biochem J 388:731–740. 10.1042/BJ20041853 15675895 PMC1183451

[B106] Taki K, Kaneko T, Mizuno N (2000) A group of cortical interneurons expressing mu-opioid receptor-like immunoreactivity: a double immunofluorescence study in the rat cerebral cortex. Neuroscience 98:221–231. 10854753 10.1016/s0306-4522(00)00124-x

[B107] Thomas F, Serratrice G, Béguin C, Aman ES, Pierre JL, Fontecave M, Laulhère JP (1999) Calcein as a fluorescent probe for ferric iron. Application to iron nutrition in plant cells. J Biol Chem 274:13375–13383. 10.1074/jbc.274.19.13375 10224100

[B108] Torti FM, Torti SV (2002) Regulation of ferritin genes and protein. Blood 99:3505–3516. 10.1182/blood.v99.10.3505 11986201

[B109] Tseng LF, Collins KA (1996) Pretreatment with pertussis toxin differentially modulates morphine- and beta-endorphin-induced antinociception in the mouse. J Pharmacol Exp Ther 279:39–46. 8858973

[B110] Wang Y, Li G, Stanco A, Long JE, Crawford D, Potter GB, Pleasure SJ, Behrens T, Rubenstein JL (2011) CXCR4 and CXCR7 have distinct functions in regulating interneuron migration. Neuron 69:61–76. 10.1016/j.neuron.2010.12.005 21220099 PMC3025760

[B111] Wang YQ, Wang MY, Fu XR, Peng Y, Gao GF, Fan YM, Duan XL, Zhao BL, Chang YZ, Shi ZH (2015) Neuroprotective effects of ginkgetin against neuroinjury in Parkinson's disease model induced by MPTP via chelating iron. Free Radic Res 49:1069–1080. 25968939 10.3109/10715762.2015.1032958

[B112] Whissell PD, Bang JY, Khan I, Xie YF, Parfitt GM, Grenon M, Plummer NW, Jensen P, Bonin RP, Kim JC (2019) Selective activation of cholecystokinin-expressing GABA (CCK-GABA) neurons enhances memory and cognition. eNeuro 6 10.1523/ENEURO.0360-18.2019 PMC6397954 30834305

[B113] White RS, Bhattacharya AK, Chen Y, Byrd M, McMullen MF, Siegel SJ, Carlson GC, Kim SF (2016) Lysosomal iron modulates NMDA receptor-mediated excitation via small GTPase, Dexras1. Mol Brain 9:38. 10.1186/s13041-016-0220-8 27080392 PMC4832449

[B114] Wilkinson J 4th, Pietsch EC, Torti SV, Torti FM (2003) Ferritin regulation by oxidants and chemopreventive xenobiotics. Adv Enzyme Regul 43:135–151. 10.1016/S0065-2571(02)00037-7 12791388

[B115] Wilkinson N, Pantopoulos K (2014) The IRP/IRE system in vivo: insights from mouse models. Front Pharmacol 5:176. 10.3389/fphar.2014.00176 25120486 PMC4112806

[B116] Wu PR, Cho KKA, Vogt D, Sohal VS, Rubenstein JLR (2017) The cytokine CXCL12 promotes basket interneuron inhibitory synapses in the medial prefrontal cortex. Cereb Cortex 27:4303–4313. 10.1093/cercor/bhw230 27497284 PMC6410508

[B117] Xu C, Fitting S (2016) Inhibition of GABAergic neurotransmission by HIV-1 tat and opioid treatment in the striatum involves µ-opioid receptors. Front Neurosci 10:497. 10.3389/fnins.2016.00497 27877102 PMC5099255

[B118] Zhou ZD, Tan EK (2017) Iron regulatory protein (IRP)-iron responsive element (IRE) signaling pathway in human neurodegenerative diseases. Mol Neurodegener 12:75. 10.1186/s13024-017-0218-4 29061112 PMC5654065

